# Increased Cardiac Risk After a Second Malignant Neoplasm Among Childhood Cancer Survivors: A FCCSS Study

**DOI:** 10.1016/j.jaccao.2023.07.008

**Published:** 2023-10-03

**Authors:** Thibaud Charrier, Nadia Haddy, Boris Schwartz, Neige Journy, Brice Fresneau, Charlotte Demoor-Goldschmidt, Ibrahima Diallo, Aurore Surun, Isabelle Aerts, François Doz, Vincent Souchard, Giao Vu-Bezin, Anne Laprie, Sarah Lemler, Véronique Letort, Carole Rubino, Stéfania Chounta, Florent de Vathaire, Aurélien Latouche, Rodrigue S. Allodji

**Affiliations:** a Cancer and Radiation Team, Centre de Research en Epidemiologie et Santé des Populations, Université Paris-Saclay– Université Paris-Sud–Université de Versailles Saint-Quentin-en-Yvelines, Villejuif, France; b Cancer and Radiation Team, Centre de Research en Epidemiologie et Santé des Populations, Institut National de la Santé et de la Recherche Médicale, Villejuif, France; c Cancer and Radiation Team, Department of Clinical Research, Gustave Roussy, Villejuif, France; d U900, Institut National de la Santé et de la Recherche Médicale, PSL Research University, Institut Curie, Saint-Cloud, France; e Department of Pediatric Oncology, Gustave Roussy, Université Paris-Saclay, Villejuif, France; f Department of Pediatric Oncology, Centre Hospitalier Universitaire, Angers, France; g Department of Radiotherapy, Centre François Baclesse, Caen, France; h Department of Supportive Care, Centre François Baclesse, Caen, France; i Department of Radiation Oncology, Gustave Roussy, Villejuif, France; j Radiothérapie Moléculaire et Innovation Thérapeutique, Institut National de la Santé et de la Recherche Médicale, Gustave Roussy, Paris-Saclay University, Villejuif, France; k SIREDO Oncology Center, Institut Curie, Paris, France; l Université Paris Cité, Paris, France; m Department of Radiation Oncology, Centre Antoine-Lacassagne, Nice, France; n Mathématiques et Informatique pour la Complexité et les Systèmes, CentraleSupélec, Université Paris-Saclay, Gif-sur-Yvette, France; o Conservatoire National des Arts et Métiers, Paris, France; p Polytechnic School of Abomey-Calavi, University of Abomey-Calavi, Cotonou, Benin

**Keywords:** additive model, anthracycline chemotherapy, cardiac disease, cardio-oncology, cumulative incidence, late effect, radiation

## Abstract

**Background:**

Childhood cancer survivors (CCS) are at an elevated risk of developing both a second malignant neoplasm (SMN) and cardiac disease.

**Objectives:**

This study sought to assess the excess of occurrence of cardiac disease after a SMN among CCS.

**Methods:**

Analyses included 7,670 CCS from the French Childhood Cancer Survivors Study cohort diagnosed between 1945 and 2000. To account for the time dependence of the occurrence of a SMN, we employed a landmark approach, considering an additive regression model for the cumulative incidence of cardiac disease. We estimated the effect of a SMN on the instantaneous risk of cardiac disease using a proportional cause-specific hazard model, considering a SMN as a time-dependent exposure. In both models, we adjusted for demographic and treatment information and considered death as a competing event.

**Results:**

In 7,670 CCS over a median follow-up of 30 years (IQR: 22-38 years), there were 378 cases of cardiac disease identified, of which 49 patients experienced a SMN. Patients who survived 25 years after their childhood cancer diagnosis and had a SMN in that time frame had a significantly increased cumulative incidence of cardiac disease, which was 3.8% (95% CI: 0.5% to 7.1%) higher compared with those without a SMN during this period. No SMN-induced excess of cardiac disease was observed at subsequent landmark times. SMNs were associated with a 2-fold increase (cause-specific HR: 2.0; 95% CI: 1.4-2.8) of cardiac disease.

**Conclusions:**

The occurrence of a SMN among CCS is associated with an increased risk of cardiac disease occurrence and risk at younger ages.

Advances in cancer treatment have significantly improved childhood cancer survival, with the 5-year survival rate exceeding 80% in most European and North American countries today.^1^ The population of childhood cancer survivors (CCS) in Europe now exceeds 300,000 people, with approximately 1 in every 1,000 individuals being a CCS.^2^ This increased population has also led to a higher rate of health issues related to late effects^3^ compared with the general population,^4^ with cardiac disease and the occurrence of a second malignant neoplasm (SMN) being among the most severe and life-threatening conditions experienced by CCS.^5^, ^6^, ^7^, ^8^

Previous studies have identified several main risk factors for cardiac disease,^9^^,^^10^ with the most important ones being cumulative doses of anthracyclines, chest radiotherapy (including heart radiation as low as 5 Gy), alkylating agents administration, and treatment at a young age. Cardiac disease has been found to contribute to excess mortality of CCS who survived breast cancer, as reported by Moskowitz et al.^11^

Despite this knowledge, the effects of a SMN on cardiac disease remain unknown. We hypothesized that the risk of cardiac disease may be influenced by both common risk factors of a SMN and cardiac disease, as well as treatments for a SMN, but whether this increased risk would translate to a higher cumulative incidence of cardiac disease among CCS is unknown. Indeed, patients who experienced a SMN have been found to have a higher mortality rate than those who did not, which could counterbalance the increased risk of cardiac disease. Therefore, in the present study, we aim to quantify both the increased risk and increased cumulative incidence of cardiac disease after a SMN among CCS.

## Methods

### Study population

The FCCSS (French Childhood Cancer Survivors Study)^12^ cohort follows 7,670 5-year survivors treated between 1945 and 2000 for solid cancer in 5 cancer centers in France. The FCCSS received approval from a National Committee on Ethics and the French National Agency Regulating Data Protection (agreement nos. 902287 and 12038829). Written informed consent was obtained from all patients, parents, or guardians in accordance with national research ethics requirements. The present analysis included all 7,670 survivors, but to address potential bias introduced by missing data, we used multiple imputation as recommended (see the Supplemental Appendix for more details).^13^

### Cancer therapy exposures

Chemotherapy, surgery, and radiotherapy information for childhood cancer treatment were abstracted from medical records. For this study, chemotherapy exposures were defined as follows: 1) receipt (or not) of any chemotherapy agent, anthracyclines, alkylating agents, or platinum agents; and 2) cumulative doses for each chemotherapy class. For each survivor, cumulative doses of each drug received per $m^{2}$ were computed by summing chemotherapy doses across cycles. Anthracycline cumulative dose was computed using doxorubicin equivalents.^14^

For external beam radiotherapy and/or brachytherapy, radiation dose distributions to the heart were retrospectively reconstructed on patient-specific voxel phantoms, considering individual patient treatment information. This information included treatment machine, type of radiation, beam energy, irradiation technique, field size and shape, gantry and collimator angles, use of accessories, target volume location, and total delivered dose. This retrospective reconstruction was necessary, because computed tomography scans were not used on many patients. More details on the methodology and dosimetry software package used have already been published.^15^ The use of voxel phantoms, in which the heart was carefully delineated, allowed the computation of the mean radiation dose.

### Outcome definitions: Identification and validation of deaths, SMN, and cardiac disease

Vital status of all patients and causes of death for deceased patients were obtained from CépiDC.^16^ Clinical and epidemiological follow-up, including self-administered questionnaires and cohort linkage with the French Hospital Database and Health Insurance Information System,^17^ was performed to identify the occurrence of iatrogenic effects. For patients treated at Gustave Roussy, clinic long-term follow-up was also performed. SMNs and cardiac diseases were identified through these different sources and subsequently validated by a trained and experienced clinical research associate. Validation was based on medical, pathology, or radiological reports obtained from the treating centers or from referring doctors, regardless of the data source used for first identification. Cardiac disease events were graded according to the Common Terminology Criteria for Adverse Events (version 4.03). In this study, we included only severe cardiac disease events (grade ≥3), based on the consideration that nonsevere cardiac disease events may be self-reported and could cause reporting bias in the data.

### Statistical analysis

**Figure 1:**
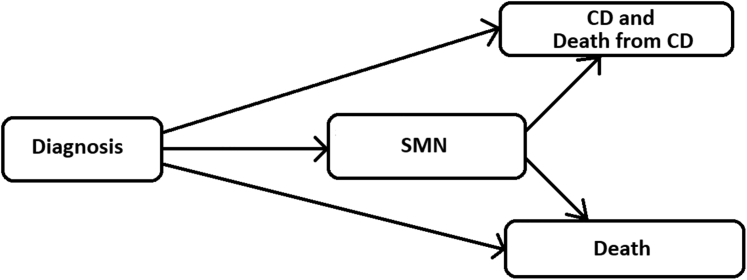
Multistate Model for an Adverse Event This diagram shows the states used for all analyses reported in this paper. All patients start in the diagnosis state and are at risk of a second malignant neoplasm (SMN), cardiac disease (CD), and death. Patients experiencing an SMN remain at risk of CD and death. Once patients experience a CD or die they are not at risk anymore.

For the study, our primary event of interest was the first occurrence of severe cardiac disease or death resulting from cardiac disease, with death from any cause considered to be a competing event. SMN was considered to be a time-dependent exposure, rather than a competing event. We defined T as the time of the first occurrence of cardiac disease or death resulting from cardiac disease (event of interest), death from other cause (competing event), or last follow-up (censor) (Figure 1). Patients (n = 35) whose first event occurred within 5 years of their childhood cancer diagnosis were excluded from the analysis.

### Cumulative incidence of cardiac disease

To estimate the effect of a SMN on cardiac disease incidence, we chose to quantify the excess in the occurrence of cardiac disease observed after a SMN, that is, the percentage of the population experiencing a cardiac disease after a SMN, not explained by non–SMN-related information. We employed a landmark analysis^18^ at 9 different time points. We included patients conditional on their survival at s∈{15,20,25,30,35} years from diagnosis and at s∈{20,25,30,35} years of age. We chose those landmark times by looking at the number of events observed after each landmark time (Supplemental Table 1) and setting 5 years between each time, which is reasonable and clinically relevant for a prediction range. Following Cortese and Andersen’s suggestion,^19^ SMN status was defined as the binary time-dependent indicator $X_{SMN}\left(s\right)=1_{\left(\text{SMN}\;\text{before}\;s\right)}$ for each landmark time. We then included $X_{SMN}\left(s\right)$ as a covariate in an additive model for the cumulative incidence of cardiac disease (Equation 1),

(1) $$P\left(T\leq t,e=cardiac\;disease|T>s,S_{MN},X\right)=F_{s,cardiac\;disease}\left(t\right)+X_{SMN}\left(s\right)\beta_{s,SMN}^{T}+X\beta_{s}^{T}$$

where $F_{s,CD}\left(t\right)=P\left(T\leq t,e=\text{cardiac}\;\text{disease}\;|T>s\right)$ is the baseline cumulative incidence function for cardiac disease, $\beta_{s}$ is the vector of effects, and X is the vector of covariates containing treatment information. The motivation for employing an additive model for the cumulative incidence of cardiac disease was due to the simplicity of interpreting the model coefficients. This model allows us to estimate the proportion of patients who would experience a cardiac disease given they experienced a SMN.

To account for the disparity of information available to clinicians when evaluating patients’ risks, we used different combinations of covariates. The most detailed combination included, among others, both radiotherapy doses to the heart and cumulative anthracyclines doses. These groupings were chosen based on previous works identifying major risk factors^9^^,^^10^ and developing risk prediction tools.^20^^,^^21^ Supplemental Table 2 summarizes the combinations of covariates considered. All treatment information used pertain to childhood cancer.

The previous regression model was estimated using pseudovalues^22^ computed using the Aalen-Johansen^23^ estimator and generalized estimating equations with identity as the link function. For regression analysis, pseudovalues are computed at a grid of time points. A small subset of time points is enough to have reliable estimates,^22^ but there must be enough events before the first grid time point and after the last. So, we have chosen the grid {2, 4, …, 20} years after landmark time.

Furthermore, we decided to perform a sensitivity analysis to determine whether the estimates of the above-mentioned method can be separated into a baseline SMN effect and a treatment of SMN effect. We performed the same analysis changing SMN information from a binary time-dependent indicator to a categorical time-dependent covariate, named $X_{SMN,type}\left(t\right)$. The levels of $X_{SMN,type}\left(t\right)$ were chosen with a clinician based on their knowledge of standard cancer treatments and their known cardiotoxicity. These levels are “no SMN,” “Breast cancer (women),” “Sarcoma, bone, soft tissue cancer,” and “Others or unknown SMN.” The levels of $X_{SMN}\left(t\right)$ are “no SMN” and “SMN.” SMN status was still determined for each landmark time.

### Cause-specific hazard of cardiac disease

To estimate the effect of an SMN on cardiac disease instantaneous risk, we chose to use an extension of the Cox model^24^^,^^25^ with considerations for competing risks. This measure has the same interpretation as the HR of the Cox model on the instantaneous risk. Of note, the effect of an SMN on the cause-specific hazard (csH) of cardiac disease cannot be directly converted into an effect of an SMN on the cumulative incidence of cardiac disease.^19^

We considered patients who survived at least 5 years after childhood cancer diagnosis and conducted the analysis using a proportional csH model, including SMN status as a time-dependent covariate $X_{SMN}\left(t\right)=1_{\left(\text{SMN}\;\text{before}\;t\right)}$.

We used 5 years since diagnosis of first cancer as the time when patients start being at risk of cardiac disease and death. Similar to the previous analysis on the additive scale for the cumulative incidence, we considered various sets of covariates to adjust our estimation of the csH, using treatment information for the childhood cancer. Those sets of covariates are detailed in Supplemental Table 2.

We performed a sensitivity analysis by categorizing SMN into 4 types. This categorization was consistent with the one used in the cumulative incidence sensitivity analysis, where we used $X_{SMN,type}\left(t\right)$ as a time-dependent covariate instead of $X_{SMN}\left(t\right)$.

All analyses were conducted using R software (version 4.2.0, R Foundation for Statistical Computing) and packages survival,^26^^,^^27^ geepack,^28^, ^29^, ^30^ and pseudo.^31^ To allow interested readers to implement the methods used, we have made the R scripts available online.^32^

## Results

Continuous data are presented as median (IQR). Cumulative incidence estimates are presented as a percentage with 95% CI. csH model results are presented as the cause-specific HR (csHR) with 95% CI.

### Descriptive analysis of the cohort

The demographic and treatment characteristics of 5-year CCS from the FCCSS cohort are presented in Table 1. Among the 7,670 survivors of cancer, 4,201 (54.8%) were female, almost half (48.6%) were younger than 5 years of age at their first childhood cancer diagnosis, and more than 80% of the cohort was still alive as of the last follow-up contact. Approximately 40% of all survivors received a combination of chemotherapy and radiation. Specifically, 31% of the population was treated with cumulative doses of anthracyclines higher than 100 mg$/m^{2}$, and more than 49% of the cohort had been exposed to heart radiation during radiotherapy.

**Table 1:** Cohort Description

Sex	
Female	4,201 (54.8%)
Male	3,469 (45.2%)
Age at diagnosis	
<5 y	3,726 (48.6%)
5-10 y	1,678 (21.9%)
10-15 y	1,623 (21.2%)
>15 y	643 (8.4%)
Deceased	
No	6,173 (80.5%)
Yes	1,497 (19.5%)
Treatment combination	
Nor radiotherapy nor chemotherapy	902 (11.8%)
Radiotherapy alone	1,088 (14.2%)
Chemotherapy alone	2,574 (33.6%)
Radiotherapy and chemotherapy	3,106 (40.5%)
Anthracyclines doses	
0 mg/m^2^	5,060 (66.0%)
0-100 mg/m^2^	209 (2.7%)
100-250 mg/m^2^	1,257 (16.4%)
>250 mg/m^2^	1,144 (14.9%)
Alkylating agent	
No	4,060 (52.9%)
Yes	3,610 (47.1%)
Platinum agent	
No	6,045 (78.8%)
Yes	1,625 (21.2%)
Mean heart RT dose	
0 Gy	3,622 (49.8%)
0-5 Gy	2,349 (32.3%)
5-15 Gy	573 (7.9%)
15-35 Gy	643 (8.8%)
>35 Gy	92 (1.3%)
Mean brain RT dose	
0 Gy	3,567 (49.0%)
0-20 Gy	2,954 (40.6%)
20-30 Gy	342 (4.7%)
30-50 Gy	409 (5.6%)
>50 Gy	7 (0.1%)
Neck RT	
No	3,609 (49.6%)
Yes	3,670 (50.4%)
Type of childhood cancer	
Unknown	10 (0.1%)
01 - Leukemias	1 (0.0%)
02 - Lymphomas	1,278 (16.7%)
03 - CNS tumor	1,140 (14.9%)
04 - Peripheral nervous tumors	1,034 (13.5%)
05 - Retinoblastomas	619 (8.1%)
06 - Renal tumors	1,140 (14.9%)
07 - Hepatic tumors	79 (1.0%)
08 - Bone sarcomas	686 (8.9%)
09 - Soft-tissue sarcomas	859 (11.2%)
10 - Germ cells and gonadal tumors	469 (6.1%)
11 - Other carcinomas	344 (4.5%)
12 - Other or unspecified tumors	11 (0.1%)

Values are n (%).

RT = radiotherapy.

Over a median follow-up (T) of 30 years (IQR: 22-38 years), 795 individuals developed an SMN, 329 developed a cardiac disease without an SMN, and 49 developed a cardiac disease after an SMN. The median time to a cardiac disease was 23 years (IQR: 15-32 years), with a median age at event of 32 years (IQR: 21-40 years). The median time to an SMN was 20 years (IQR: 13-29 years), with a median age at event of 29 years (IQR: 19-38 years). The majority of the population with an SMN was treated more recently than 2000: only 6% (n = 45) were diagnosed before 1980, 11% (n = 90) between 1980 and 1989, and 22% (n = 177) between 1990 and 1999, while 61% (n = 483) were diagnosed after 2000. The number of patients included at each landmark time and the number of observed events are listed in Supplemental Table 1.

The identified cardiac diseases among those with and without an SMN included heart failure (n = 194 [51.3%]), valvular heart disease (n = 55 [14.6%]), arrhythmia (n = 46 [12.2%]), pericardial disease (n = 28 [7.4%]), ischemic heart disease (n = 28 [7.4%]), and other heart diseases (n = 27 [7.1%]). For SMN, all malignant neoplasms were included. Of the 795 SMNs, the most common ones identified were breast cancer (n = 90 [11.3%]), thyroid cancer (n = 82 [10.3%]), bone cancer (n = 70 [8.8%]), and skin epitheliomas and carcinoma (n = 68 [8.5%]). A fourth of post-SMN cardiac diseases occurred after breast cancer (Supplemental Table 3).

**Figure 2:**
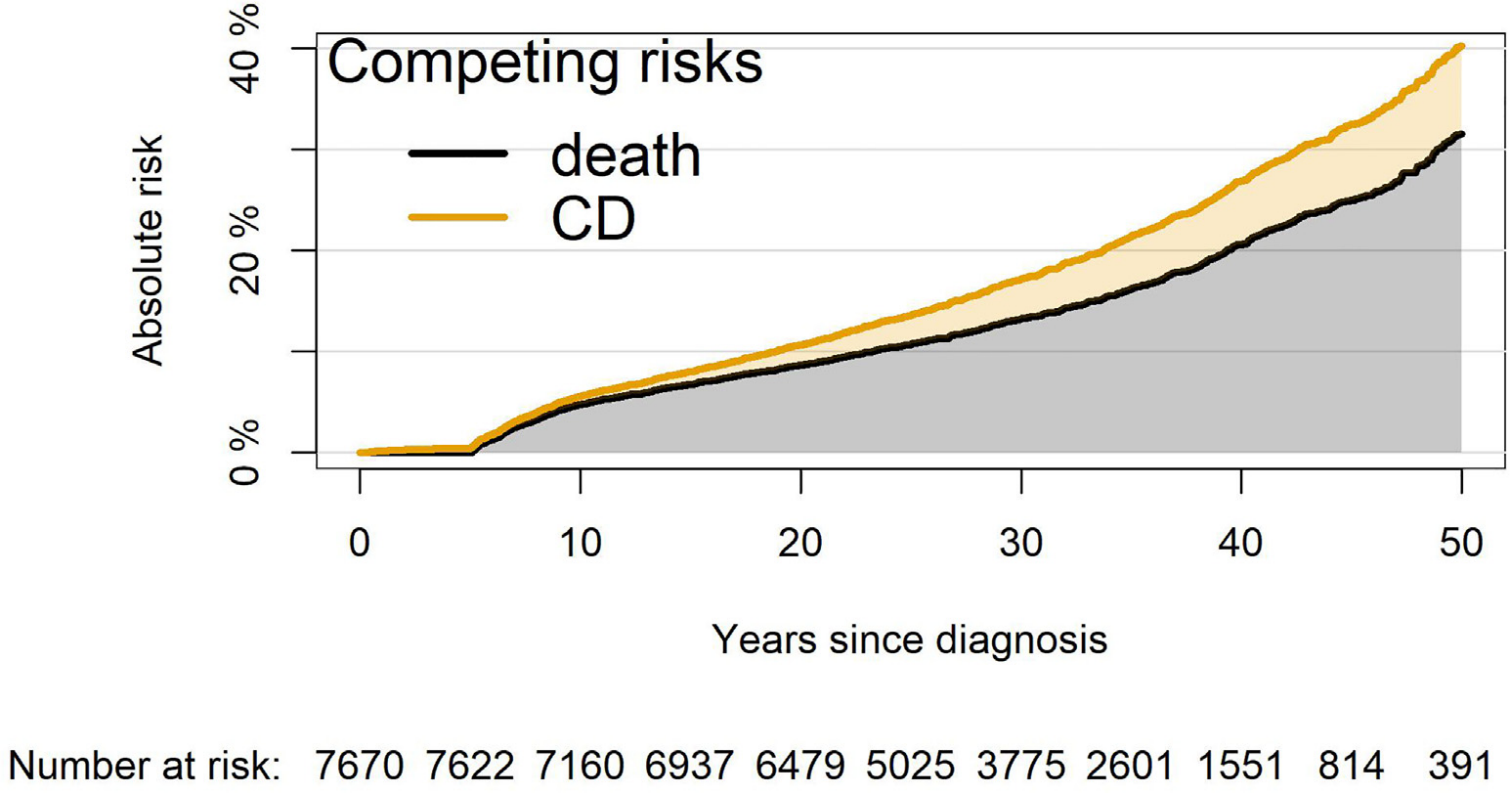
Stacked Cumulative Incidence Plot of Death and CD This plot represents our cohort of 7,670 5-year childhood cancer survivors, illustrating that within 50 years of their childhood cancer diagnosis, 30% die and 8% experience a cardiac disease (CD). This risk is observed regardless of the occurrence of a second malignant neoplasm. The plot highlights that this population is at a high risk of both death and CDs.

The cumulative incidence of cardiac disease was 3.9% (95% CI: 3.4% to 4.3%) at 30 years after diagnosis and 8.4% (95% CI: 7.3% to 9.5%) at 50 years after diagnosis for the whole cohort, presented in Figure 2. For patients treated with both radiotherapy (any dose) and chemotherapy (any agent), the cumulative incidence of cardiac disease was 5.8% (95% CI: 4.9% to 6.7%) at 30 years after diagnosis and 12.0% (95% CI: 10.2% to 13.8%) at 50 years after diagnosis (Supplemental Figure 1). In comparison, for patients treated with neither radiotherapy nor chemotherapy, the cumulative incidence was 1.7% (95% CI: 0.2% to 3.3%) at 50 years after diagnosis.

### SMN effect on the cumulative incidence of cardiac disease: Additive regression model

**Figure 3:**
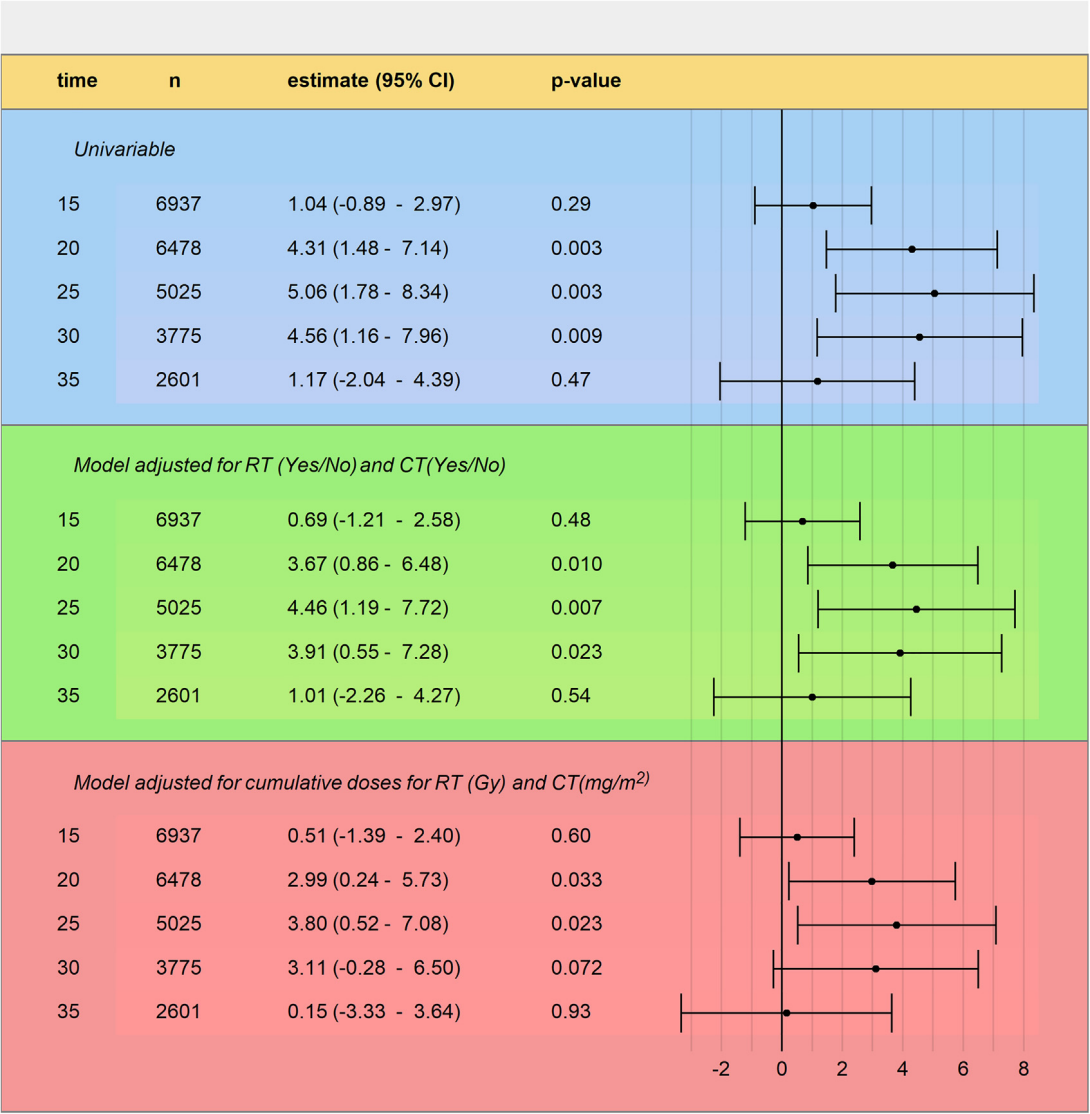
Additive Effect of SMN on Cumulative Incidence of Cardiac Disease This plot summarizes the effect of a second malignant neoplasm (SMN) on the cumulative incidence of cardiac disease. Using an additive model on the cumulative incidence, combined with a landmark strategy, we determined that among patients surviving 25 years after diagnosis, experiencing an SMN was associated with an increased cumulative incidence of cardiac disease of 3.8%. We used time since childhood cancer diagnosis and included death as a competing event. Multivariable models were also adjusted for sex, age, and year of childhood cancer diagnosis. CT = chemotherapy; RT = radiotherapy.

The cumulative incidence of cardiac disease was higher when an SMN occurred for landmark times 20, 25, and 30 years after diagnosis, as well as 30 and 35 years of attained age. The maximum estimated excess was 3.8% (95% CI: 0.5% to 7.1%) for the landmark time of 25 years after diagnosis (Figure 3). Note that, here, estimates correspond to whether and how much the probability of experiencing cardiac disease changes between SMN survivors and baseline CCS. The Central Illustration, Figure 3, and Table 2 summarize the estimated excess of cumulative incidence of cardiac disease after an SMN for each configuration. As detailed in Supplemental Table 1, the 3.8% excess for landmark time 25 years after diagnosis translated into approximately 10 cardiac diseases (259 patients with an SMN at this time, 259 · 3.8 / 100 = 9.8) out of the 30 expected after an SMN (259 patients with an SMN at this time, 11.5% cumulative incidence of cardiac disease, 259 · 0.115 = 29.8) in this category.

**Central Illustration:**
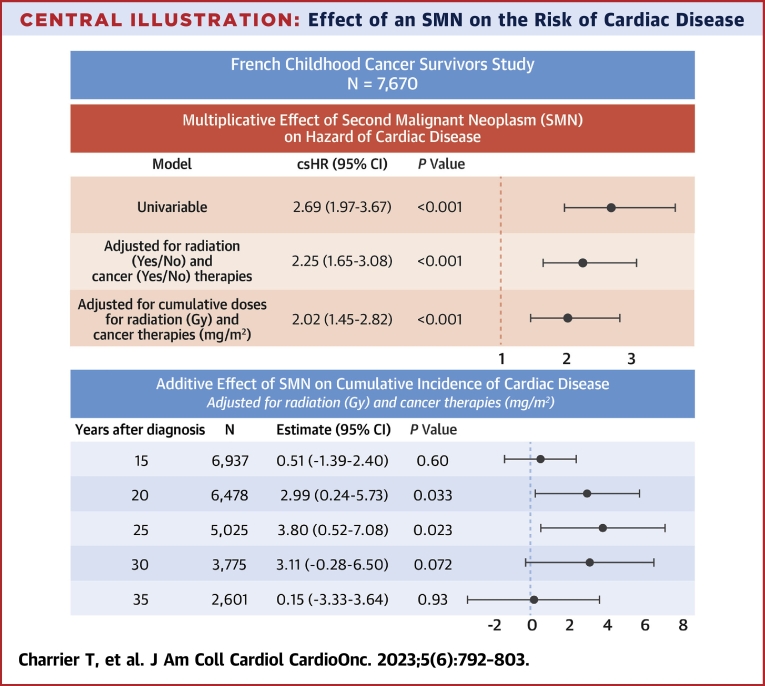
Effect of an SMN on the Risk of Cardiac Disease Using a proportional cause-specific hazard model, we determined a 2-fold increase in the risk of cardiac disease after a second malignant neoplasm (SMN). We also used an additive model on the cumulative incidence, combined with a landmark strategy, and determined that among patients surviving 25 years after diagnosis, experiencing an SMN was associated with an increased cumulative incidence of cardiac disease of 3.8%. We used time since childhood cancer diagnosis and included death as a competing event. Multivariable models were also adjusted for sex, age and year of childhood cancer diagnosis.

**Table 2:** Estimation of the Additive Effect of an SMN Occurrence on the Cumulative Incidence of Cardiac Disease

Attained Age (y)	Covariate	β_SMN_ (95% CI)	*P* Value
20	Univariable	0.62 (−1.23 to 2.46)	0.51
	Model adjusted for RT (yes/no) and CT (yes/no)^a^	0.29 (−1.55 to 2.13)	0.76
	Model adjusted for cumulative doses for RT (Gy) and CT (mg/m^2^)^b^	0.50 (−1.30 to 2.29)	0.59
25	Univariable	2.33 (−0.19 to 4.84)	0.070
	Model adjusted for RT (yes/no) and CT (yes/no)^a^	2.04 (−0.46 to 4.55)	0.11
	Model adjusted for cumulative doses for RT (Gy) and CT (mg/m^2^)^b^	1.88 (−0.56 to 4.33)	0.14
30	Univariable	3.87 (0.86 to 6.88)	0.012
	Model adjusted for RT (yes/no) and CT (yes/no)^a^	3.49 (0.52 to 6.47)	0.021
	Model adjusted for cumulative doses for RT (Gy) and CT (mg/m^2^)^b^	3.17 (0.22 to 6.11)	0.035
35	Univariable	4.39 (1.09 to 7.68)	0.009
	Model adjusted for RT (yes/no) and CT (yes/no)^a^	4.01 (0.76 to 7.26)	0.016
	Model adjusted for cumulative doses for RT (Gy) and CT (mg/m^2^)^b^	3.25 (0.01 to 6.49)	0.049

Death is included as a competing event. The time scale is attained patient age. For patients who survived ≥35 years of age, the univariable analysis shows that patients experiencing an SMN had a cumulative incidence 4.4% (95% CI: 1.1%-7.7%) higher than those who did not have an SMN. When adjusting for age at first diagnosis, RT (yes/no), and chemotherapy (yes/no), the cumulative incidence of cardiac disease was 4.0% (95% CI: 0.8%-7.3%) higher when an SMN occurred before 35 years of age. When including all treatment information (age at diagnosis, sex, year of diagnosis, average RT dose at the heart and brain, exposure of the neck to RT, cumulative anthracycline dose, use of alkylating agent), the cumulative incidence of cardiac disease was 3.3% (95% CI: 0.0%-6.5%) higher when an SMN occurred before 35 years of age.

CT = chemotherapy; RT = radiotherapy; SMN = second malignant neoplasm.

a Adjusted on sex, age at diagnosis, cumulative anthracyclines doses, alkylating agent (yes/no), mean RT dose at the heart, mean RT dose at the brain, RT at the neck (yes/no), and the year of first cancer diagnosis (before/after 1980).

b Adjusted on RT (yes/no), chemotherapy (yes/no), sex, age at diagnosis, and the year of first cancer diagnosis (before/after 1980).

For the landmark times 15 and 35 years after diagnosis, as well as 20 and 25 years of attained age, no significant excess of cardiac disease incidence due to an SMN was observed. In the univariable analysis for patients who survived ≥25 years after diagnosis (Figure 3, Central Illustration), those experiencing an SMN had a cumulative incidence 5.1% (95% CI: 1.8% to 8.3%) higher than those who did not have an SMN. When adjusting for age at first diagnosis, radiotherapy (yes/no), and chemotherapy (yes/no), the cumulative incidence of cardiac disease was 4.5% (95% CI: 1.2% to 7.7%) higher when an SMN occurred in the first 25 years after childhood cancer diagnosis. When including all treatment information (age at diagnosis, sex, year of diagnosis, average radiotherapy dose at the heart and brain, exposure of the neck to radiotherapy, cumulative anthracycline dose, use of alkylating agent), the cumulative incidence of cardiac disease was 3.8% (95% CI: 0.5% to 7.1%) higher when an SMN occurred in the first 25 years after childhood cancer.

Results on the attained age time scale were similar. In the univariable analysis for patients who survived ≥35 years of age (Table 2), those experiencing an SMN had a cumulative incidence 4.4% (95% CI: 1.1% to 7.7%) higher than those who did not have an SMN. When adjusting for age at first diagnosis, radiotherapy (yes/no), and chemotherapy (yes/no), the cumulative incidence of cardiac disease was 4.0% (95% CI: 0.8% to 7.3%) higher when an SMN occurred before 35 years of age. When including all treatment information (age at diagnosis, sex, year of diagnosis, average radiotherapy dose at the heart and brain, exposure of the neck to radiotherapy, cumulative anthracycline dose, use of alkylating agent), the cumulative incidence of cardiac disease was 3.3% (95% CI: 0.0% to 6.5%) higher when an SMN occurred before 35 years of age.

When categorizing the SMN status into 4 types, we did not observe any statistically significant effect, due to high standard errors. However, we did observe tendencies in the pointwise estimates (Supplemental Figure 2) among the cancer types. When adjusting for all treatment information, we observed that breast cancer (10.9%; 95% CI: −12.7% to 34.5%); sarcoma, bone, or soft tissue cancer (7.7%; 95% CI: −1.2% to 16.6%); and other SMN types (2.0%; 95% CI: −1.0% to 5.0%) showed an increase in cardiac disease cumulative incidence when an SMN occurred before the patient was 30 years of age, though not all were statistically significant.

To better understand the importance of those quantities, we provided the cumulative incidence curves for each landmark time in Supplemental Figure 3.

Following recommendations for the reporting of competing risks results, the effects of an SMN on cumulative incidences of death are provided in Supplemental Figures 4 and 5, and cumulative incidences conditional on survival time are provided in Supplemental Figure 6. The effect of an SMN on the cumulative incidence of death varies with time and by cancer type, with earlier times having the highest increase (17.5%; 95% CI: 11.3% to 23.7%) for patients who survived up to 20 years of age. The cumulative incidences of death conditional on survival at 15, …, 35 years after childhood cancer diagnosis (Supplemental Figure 6) show that older patients have a death rate slightly higher than younger patients, while remaining comparable.

### SMN effect on the csH of cardiac disease

For all combinations of treatment-related risk factors, the occurrence of an SMN had a deleterious effect on the csH of cardiac disease (Central Illustration, Supplemental Figure 7). In univariable analysis, the occurrence of an SMN resulted in a 3-fold increase in the hazard of cardiac disease (csHR: 2.7; 95% CI: 2.0 to 3.7). When adjusting for age at diagnosis, radiotherapy (yes/no), and chemotherapy (yes/no), the occurrence of an SMN caused a 2-fold increase in the hazard of cardiac disease (csHR: 2.3; 95% CI: 1.6 to 3.1). When including all treatment information (age at diagnosis, sex, year of diagnosis, average radiotherapy dose at the heart and brain, exposure of the neck to radiotherapy, cumulative anthracycline dose, use of alkylating agent, use of platinum agent), the occurrence of an SMN increased the likelihood of cardiac disease (csHR: 2.0; 95% CI: 1.5 to 2.8). Using detailed doses for chemotherapy and radiotherapy doses instead of binary information did not modify the estimation of the effect of an SMN on the csH of cardiac disease.

When categorizing the SMN status into 4 types, we observed some differences (Supplemental Figure 8). In univariable analysis, the occurrence of breast cancer (csHR: 3.5; 95% CI: 2.1 to 5.8); sarcoma, bone, or soft tissue cancer (csHR: 3.2; 95% CI: 1.6 to 6.2); and other SMN (csHR: 1.9; 95% CI: 1.5 to 2.6) was associated with an increased risk of cardiac disease. When adjusting for all treatment information (same as previous), the occurrence of breast cancer (csHR: 1.9; 95% CI: 1.1 to 3.2); sarcoma, bone, or soft tissue cancer (csHR: 2.6; 95% CI: 1.3 to 5.0); and other SMN (csHR: 1.4; 95% CI: 1.1 to 2.0) also was associated with an increased hazard of cardiac disease. HR estimates for the competing risk of death overall and by SMN type are provided in Supplemental Figures 9 and 10, respectively.

### Treatment effects

Although this study focused on estimating the effect of an SMN and did not aim to estimate treatment effects, we did obtain csHR estimates for specific covariates while estimating the effect of an SMN (Supplemental Table 4). Among the risk factors analyzed, the most important ones were a >5 Gy average heart dose, >250 mg/ $m^{2}$ cumulative dose of anthracycline, and the use of alkylating agents, which align with findings of previous studies.^9^^,^^10^ As previously determined,^33^ we did not find an increase of cardiac disease risk for <100 mg/m^2^ cumulative anthracycline dose.

## Discussion

### Cardiac diseases among CCS

In this study, we followed a well-defined population of CCS (FCCSS) over an extended treatment period spanning from 1946 to 2000. Our data collection involved a comprehensive approach, including self-reported questionnaires, hospital-based databases/registries, and clinically assessed data from survivors participating in long-term clinical follow-ups. We observed a deleterious effect of SMNs on the csH scale and the cumulative incidence of cardiac disease among CCS. We conclude that CCS who experienced an SMN had higher instantaneous and absolute risks of severe cardiac disease compared with those who did not experience an SMN. To the best of our knowledge, this is the first study to explore severe cardiac disease occurrence after experiencing an SMN among CCS. Previous research has identified that CCS are at high risk of multimorbidity,^4^ which is consistent with our results. This study was a first attempt to understand in depth the relationship between the multimorbidity among CCS, specifically an SMN and severe cardiac disease, 2 of the most frequent and important life-threatening adverse events. In our population of 5-year CCS, the cumulative incidence of severe cardiac disease was 3.9% (95% CI: 3.4% to 4.3%) and 8.4% (95% CI: 7.3% to 9.5%) at 30 and 50 years of age, respectively. These proportions closely align with the 30-year cumulative incidence of first severe cardiac disease in the Netherlands Cancer Institute study (4.2%; 95% CI: 2.8% to 5.6%)^34^ and the Childhood Cancer Survivor Study (CCSS) across 27 participating institutions in the United States and Canada (4.8%; 95% CI: 4.3% to 5.2%).^35^

It is well known that both heart radiation and anthracycline-containing chemotherapy can increase the risk of severe cardiac disease in CCS.^10^^,^^35^ Our findings concur with these previous studies and support current surveillance guidelines that take into account cardiotoxicity from all cancer treatments for risk stratification. Our results also suggest a possible increase in cardiac risk after an SMN. A major strength of this study is the access to a cohort of CCS with long-term follow-up and detailed clinical history, including information on both anthracycline doses and radiotherapy doses at the heart. This allowed us to adjust for multiple configurations of childhood cancer treatment and explore the evolution of the excess of severe cardiac disease after an SMN over time. However, the small number of severe cardiac diseases after an SMN somewhat limits this strength. It will be useful to conduct the proposed analysis on larger cohorts, such as Pancare^36^ or the CCSS.^37^

Many cardiotoxic childhood cancer treatments are also known to increase the risk of an SMN. We have detailed records available, allowing us to adjust on important factors, such as radiotherapy, chemotherapy, and age at childhood cancer. We observed the expected effect of these factors on cardiac risk, and the estimated effect of an SMN decreased when adjusting for them, which is consistent with previous results indicating shared risk factors for both an SMN and cardiac disease.

We observed an increase in the likelihood of cardiac disease at all times, while the effect on the cumulative incidence was time-dependent (Figure 3, Central Illustration). This difference may be explained by the increased risk of death of older patients after an SMN (Supplemental Figure 6), which can compensate for the increased risk of cardiac disease. Indeed, if the effect on the risk of cardiac disease remains stable but the instantaneous risk of death increases, the effect on the cumulative incidence will decrease, possibly disappearing or even reversing. This does not mean that CCS who experience an SMN at an older age have a better prognosis than those who are diagnosed earlier; rather, their death rate becomes so high that cardiac diseases become less relevant. The evolution of the effect of an SMN on the cumulative incidence of cardiac disease may also be influenced by the overall increase of cardiac disease at a later age, possibly reducing the excess of cardiac disease caused by an SMN, as patients may have had a cardiac disease with or without an SMN.

Finally, it is possible that an effect may exist but we may have not detected it due to the small number of patients followed for an extended period and the small number of observed events. In the latter case, larger cohorts could induce clearer results. The difference observed for younger patients could be explained by their more recent diagnosis of an SMN (Supplemental Figure 11), resulting in a higher death rate (Supplemental Figure 4) compared with patients with longer survival periods.

### Study limitations

The lack of SMN treatment data precluded a more robust analysis of the associations of SMN treatments and other factors with the risk of cardiac disease. This information was unavailable for most of our cohort, as patients were treated at different hospitals for their SMN than for their childhood cancer. However, we did have access to the type of SMN, which was used as a proxy for SMN treatments. Due to a small number of events, we cannot draw conclusive effects of each SMN type on the cumulative incidence (Supplemental Figure 2), although point estimates suggest there may be differences. Results on the csH scale (Supplemental Figure 8) are more conclusive and show that sarcoma, bone, and soft tissue SMN induce the highest increase. This suggests that cardiotoxic SMN treatments are contributing to the increase of cardiac risk after an SMN. Aside from the cardiotoxicity of those treatments, unobserved shared risk factors of an SMN and cardiac disease may exist, and they could explain the increased cardiac risk observed for the category “Others or unknown” SMN. The effects of these factors are uncertain pending data on SMN treatment doses, which are required to conduct a mediation analysis. Another explanation could be a potential effect of having an SMN, apart from cardiotoxic SMN therapy, which may be related to an overall increased frailty of the patients. Finally, some detection bias is likely to occur due to increased care provided to SMN patients during and after their SMN treatment. To minimize this bias, we considered only cardiac events of grade 3 or higher, but this may not have fully removed the bias. We suggest that the excess of cardiac disease after an SMN is partially due to cardiotoxicity introduced by the SMN treatment, because the mentioned biases are unlikely to account for the full differences between cancer types.

The estimates by cancer type may be imprecise (large CI) due to the low number of observed events. Therefore, similar analyses in a larger cohort (Pancare, CCSS)^36^^,^^37^ may help detect differences by cancer type. Further work, including the collection of SMN treatment dose data, would be necessary to better quantify the extent to which the increase is due to SMN treatment, identify the treatments that contribute the most to the increased occurrence of cardiac disease, and investigate if the occurrence of an SMN increases cardiac risk in a way unrelated to the cardiotoxicity of treatments.

## Conclusions

Our study shows that CCS diagnosed with an SMN have both a higher csH of cardiac disease and cumulative incidence of cardiac disease, likely related to the cardiotoxicity of SMN treatments. This finding is important because, although childhood cancer survival rates increased through the improvement of modern therapies, this success carries an increased risk of late effects, including SMNs and cardiac disease. Therefore, our findings provide important insights for clinical practice guidelines concerning SMNs and cardiac disease post-therapy surveillance and risk-reducing strategies.

Perspectives **COMPETENCY IN MEDICAL KNOWLEDGE:** CCS experiencing an SMN are at increased risk of cardiac disease, with sarcoma, bone, soft tissue, and breast cancer survivors being at a higher risk.

**TRANSLATIONAL OUTLOOK:** Further research is needed to assess SMN treatment effects and whether or not there is a baseline effect unrelated to treatments.

## Funding Support and Author Disclosures

This work was supported and funded by the Gustave Roussy Foundation (Pediatric Program "Guérir le Cancer de l’Enfant"), the ITMO (Instituts thématiques multiorganismes) Cancer d’Aviesan Program (RadioPrediTool project no. 20CM112-00), the INCa (Institut national du cancer)/ARC (Foundation ARC for Cancer Research) foundation (CHART project), the Foundation ARC for Cancer Research (grant no. Pop-HaRC 201401208), the "START" PAIR Research Program (grant no. INCa-Fondation ARC-LNCC 11902), and the Ligue Nationale Contre le Cancer association. These funding agencies had no role in the design and conduct of the study, in the collection, management, analysis, and interpretation of the data, or in the preparation, review, and approval of the manuscript. The authors have reported that they have no relationships relevant to the contents of this paper to disclose.
