# Supplementary material for: Increased Cardiac Risk After a Second Malignant Neoplasm Among Childhood Cancer Survivors: A FCCSS Study
Source: JACC CardioOncol. 2023 Oct 3;5(6):792–803. doi: 10.1016/j.jaccao.2023.07.008 (PMC10774765; doi:10.1016/j.jaccao.2023.07.008)
Supplement: Supplemental Appendix [file mmc1.docx]

#

# Appendix

**Increased Cardiac Risk After a Second Malignant Neoplasm Among Childhood Cancer Survivors, a FCCSS Study**

Table of Contents:

- Page 3-4: Multiple Imputation
- Page 5-6: Supplemental Table 1: [Number of Patients at Risk, and Number of Events Observed for Each Landmark Time]
- Page 7-8: Supplemental Table 2: [Description of Covariates Used for Prognosis]
- Page 9-10: Supplemental Table 3: [Number of CD After Each SMN]
- Page 11-14**:** Supplemental Table 4: [Effect of Treatments on the Cause Specific Hazard of CD.]
- Page 15: Supplemental Figure 1: [Cumulative Incidence plot of CD conditional on childhood cancer treatment.]
- Page 16-17: Supplemental Figure 2: [Additive effect of SMN on the Cumulative Incidence of CD]
- Page 18: Supplemental Figure 3: [Cumulative Incidence of CD for CCS at each landmark time.]
- Page 19: Supplemental Figure 4: [Additive effect of SMN on the Cumulative Incidence of death]
- Page 20-21: Supplemental Figure 5: [Additive effect of SMN on the Cumulative Incidence of death.]
- Page 22: Supplemental Figure 6: [Cumulative Incidence of death for CCS at each landmark time.]
- Page 23: Supplemental Figure 7: [Multiplicative effect of SMN on the risk of CD.]
- Page 24-25: Supplemental Figure 8: [Multiplicative effect of SMN on the risk of CD.]
- Page 26: Supplemental Figure 9: [Multiplicative effect of SMN on the risk of death.]
- Page 27-28: Supplemental Figure 10: [Multiplicative effect of SMN on the risk of death.]
- Page 29: Supplemental Figure 11: [Distribution of SMN times for each landmark time]

# Multiple Imputation

Following STRATOS recommendations, we used multiple imputation to replace our missing data. All our missing data are radiotherapy dosimetry, as a consequence of the lack of information required to compute them. For external beam radiotherapy and/or brachytherapy, more details on the methodology and dosimetry software package used have already been published 14 & Vu-Bezin e al. Our dose reconstruction requires three main types of input data: imaging of patient anatomy, treatment plan and radiation source characterization. Radiation dose distributions to the anatomical region of interest (heart, brain or neck) were retrospectively reconstructed on patient-specific voxel phantoms, taking into account individual patient treatment information. These included treatment machine, type of radiation, beam energy, irradiation technique, field size and shape, gantry and collimator angles, use of accessories, target volume location, and total delivered dose. For patients treated before the computed tomography (CT) scan era or when CT scans were not available, a full anthropomorphic voxel phantom was used. In addition, because the whole-body CT scans were not available, the phantoms were also needed for anatomic regions not included in CT scans. The use of voxel phantoms, in which the heart, brain and neck were carefully delineated, allowed the computation of the mean radiation doses

We had missing dosimetry at the heart, neck, and brain for 391. The FCCSS follows 7,670 patients, 4194 of whom have been exposed to radiotherapy as a first treatment. Our proportion of missing radiotherapy dosimetry data is therefore 6.9% of the full cohort for the neck (12.6% of those exposed to radiotherapy) and 3.8% of the full cohort for the brain and heart (7.0% of those exposed to radiotherapy).

We used the R^25^ package mice^13^ to both impute the missing values and pool the results from our analysis. To predict the missing values, we used the covariates age at childhood cancer diagnosis, year of childhood cancer diagnosis, use of any chemotherapy, anthracycline cumulative dose, use of alkylating or platinum agent, sex, type of first cancer, and other imputed radiotherapy doses. We did impute the category ($0$ Gy, $0-5$ Gy, …) and not the average dose at the organ because we use only the categorical version in our models.

When comparing results to the use of a “Missing” label, results were stable. This was expected, because of our small number of missing values.

#

# Supplemental Tables

**Supplemental Table 1**: **Number of Patients at Risk, and Number of Events Observed for Each Landmark Time**

| landmark time | number of patients | Observed SMN | Observed Cardiac Disease (after SMN) | Cumulative Incidence of Cardiac Disease, given SMN before landmark time | Cumulative Incidence of Cardiac Disease, given SMN free at landmark time |
| --- | --- | --- | --- | --- | --- |
| **Years Since Diagnosis** | | | | |  |
| 15 | 6937 | 177 | 282 (13) | 10.4 | 4.2 |
| 20 | 6478 | 258 | 226 (21) | 14.8 | 4.5 |
| 25 | 5025 | 259 | 168 (20) | 11.5 | 4.9 |
| 30 | 3775 | 261 | 115 (19) | 10.5 | 5.3 |
| 35 | 2601 | 224 | 64 (8) | 6.4 | 5.6 |
| **Attained Age** | | | | |  |
| 20 | 7057 | 154 | 292 (9) | 5.3 | 3.6 |
| 25 | 6275 | 199 | 245 (16) | 9.9 | 4.6 |
| 30 | 5381 | 221 | 203 (19) | 14.3 | 5.4 |
| 35 | 4335 | 265 | 144 (20) | 13.2 | 5.8 |

**Supplemental Table 2**: **Description of Covariates Used for Prognosis**

|  | Univariable^1^ | Model adjusted for RT (Yes/No) and CT (Yes/No) ^1^ | Model adjusted for RT (Yes/No) and cumulative doses for CT^2^ | Model adjusted for CT (Yes/No) and cumulative doses for RT^2^ | Model adjusted for cumulative doses for RT (Gy) and CT(mg/m^2^)^1^ |
| --- | --- | --- | --- | --- | --- |
| SMN | True | True | True | True | True |
| Sex |  | True | True | True | True |
| RT |  | True | True |  |  |
| CT |  | True |  | True |  |
| Age at childhood cancer diagnosis |  | True | True | True | True |
| Year of childhood cancer diagnosis |  | True |  |  | True |
| Cum. Anthracycline doses |  |  | True |  | True |
| Alkylating agent |  |  | True |  | True |
| Platinum agent |  |  | True |  | True |
| Mean RT Doses @ Heart |  |  |  | True | True |
| Mean RT Doses @ Brain |  |  |  |  | True |
| RT @ Neck |  |  |  |  | True |

1. Included in the main analysis.
2. Included only in the appendix analysis.

**Supplemental Table 3**: **Number of Cardiac Diseases After Each SMN**

| Type of SMN | SMN (n) | SMN (%) | Cardiac Disease (n) | Post-SMN Cardiac Disease (%) | Survivors Cardiac Disease (%) |
| --- | --- | --- | --- | --- | --- |
| Breast : women | 90 | 11.3 | 12 | 24.5 | 13 |
| Unknown Cancer Type | 97 | 12.2 | 9 | 18.4 | 9 |
| Skin : epitheliomas and carcinoma | 89 | 11.2 | 6 | 12.2 | 7 |
| Thyroid | 82 | 10.3 | 4 | 8.2 | 5 |
| Malignant skin melanoma | 17 | 2.1 | 4 | 8.2 | 24 |
| Bone | 70 | 8.8 | 3 | 6.1 | 4 |
| Soft tissue | 43 | 5.4 | 2 | 4.1 | 5 |
| Colon rectum | 22 | 2.8 | 2 | 4.1 | 9 |
| Buccal cavity | 15 | 1.9 | 2 | 4.1 | 13 |
| Leukemia | 23 | 2.9 | 1 | 2.0 | 4 |
| Kidney and urinary annexes | 17 | 2.1 | 1 | 2.0 | 6 |
| Liver | 10 | 1.3 | 1 | 2.0 | 10 |
| Other respiratory system | 6 | 0.8 | 1 | 2.0 | 17 |
| Hodgkin disease | 2 | 0.3 | 1 | 2.0 | 50 |
| Other | 97 | 12.2 | 0 | 0.0 | 0 |
| Brain : meningiomas | 58 | 7.3 | 0 | 0.0 | 0 |
| Brain and CNS (except meningiomas) | 57 | 7.2 | 0 | 0.0 | 0 |

**Supplemental Table 4**: **Effect of Treatments on the Cause Specific Hazard of** C**ardiac Disease** . The effect is multiplicative. This table shows the effect of known risk factors of cardiac disease, to help the interpretation of the effect of SMN. It also shows consistencies with other studies.

| covariates | term | labs | estimate | P value |
| --- | --- | --- | --- | --- |
| Model adjusted for RT (Yes/No) and CT(Yes/No) | RT | Yes | 1.89 | <0.001 |
|  | CT | Yes | 3.07 | <0.001 |
|  | Sex | Female | 1.12 | 0.31 |
|  | Age at diagnosis | 5-10 y | 1.51 | 0.003 |
|  |  | 10-15 y | 1.59 | <0.001 |
|  |  | > 15 y | 2.3 | <0.001 |
|  | Year of first cancer diagnosis | After 1980 | 0.59 | <0.001 |
| Model adjusted for RT (Yes/No) and cumulative doses for CT | RT | Yes | 2.33 | <0.001 |
|  | Sex | Female | 1.08 | 0.50 |
|  | Age at diagnosis | 5-10 y | 1.23 | 0.14 |
|  |  | 10-15 y | 1.19 | 0.24 |
|  |  | > 15 y | 1.60 | 0.025 |
|  | Anthracyclines doses | 0-100 mg/m2 | 0.73 | 0.54 |
|  |  | 100-250 mg/m2 | 1.33 | 0.16 |
|  |  | > 250 mg/m2 | 3.33 | <0.001 |
|  | Alkylating agent | Yes | 1.33 | 0.062 |
|  | Platinum agent | Yes | 0.78 | 0.16 |
| Model adjusted for CT (Yes/No) and cumulative doses for RT | CT | Yes | 2.60 | <0.001 |
|  | Sex | Female | 1.01 | 0.91 |
|  | Age at diagnosis | 5-10 y | 1.07 | 0.60 |
|  |  | 10-15 y | 1.04 | 0.77 |
|  |  | > 15 y | 1.43 | 0.061 |
|  | Mean heart RT dose | 0-5 Gy | 1.17 | 0.27 |
|  |  | 5-15 Gy | 1.64 | 0.011 |
|  |  | 15-35 Gy | 4.90 | <0.001 |
|  |  | >35 Gy | 11.22 | <0.001 |
| Model adjusted for cumulative doses for RT (Gy) and CT(mg/m^2^) | Sex | Female | 1.06 | 0.62 |
|  | Age at diagnosis | 5-10 y | 1.08 | 0.58 |
|  |  | 10-15 y | 0.93 | 0.65 |
|  |  | > 15 y | 1.25 | 0.31 |
|  | Anthracyclines doses | 0-100 mg/m2 | 0.59 | 0.29 |
|  |  | 100-250 mg/m2 | 1.26 | 0.25 |
|  |  | > 250 mg/m2 | 4.03 | <0.001 |
|  | Alkylating agent | Yes | 1.29 | 0.060 |
|  | Platinum agent | Yes | 1.18 | 0.39 |
|  | Mean brain RT dose | 0-20 Gy | 1.57 | 0.29 |
|  |  | 20-30 Gy | 1.06 | 0.91 |
|  |  | 30-50 Gy | 0.41 | 0.15 |
|  |  | >50 Gy | 0.00 | <0.001 |
|  | Mean heart RT dose | 0-5 Gy | 2.35 | 0.016 |
|  |  | 5-15 Gy | 4.39 | <0.001 |
|  |  | 15-35 Gy | 13.59 | <0.001 |
|  |  | >35 Gy | 34.35 | <0.001 |
|  | Neck RT | Yes | 0.32 | 0.051 |
|  | Year of first cancer diagnosis | After 1980 | 0.67 | 0.004 |

b ' Model adjusted for RT (Yes/No) and CT(Yes/No)’ is adjusted on radiotherapy (yes/no), chemotherapy (yes/no), Sex, age at diagnosis, and the year of first cancer diagnosis (before/after 1980).
c ' Model adjusted for RT (Yes/No) and cumulative doses for CT ' is adjusted on radiotherapy (yes/no), Sex, Age at Diagnosis, cumulative anthracyclines doses and alkylating agent (yes/no).
d ' Model adjusted for CT (Yes/No) and cumulative doses for RT ' is adjusted on chemotherapy (yes/no), Sex, Age at Diagnosis and average radiotherapy dose at the heart.
e ' Model adjusted for cumulative doses for RT (Gy) and CT(mg/m2)' is adjusted on Sex, Age at Diagnosis, cumulative anthracyclines doses, alkylating agent (yes/no), mean radiotherapy dose at the heart, mean radiotherapy dose at the brain , radiotherapy at the neck (yes/no) and the year of first cancer diagnosis (before/after 1980).

#

# Stratified Cumulative Incidence


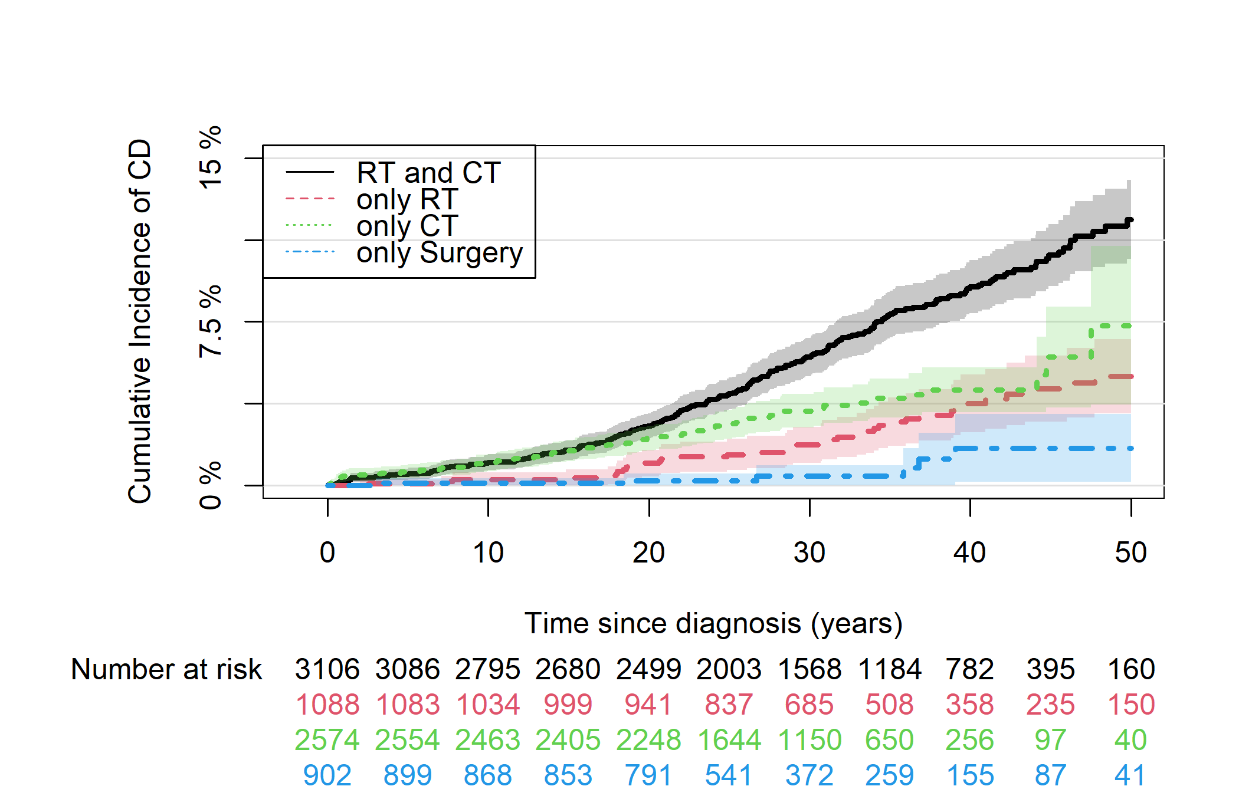


**Supplemental Figure 1**: **Cumulative Incidence plot of cardiac disease conditional on childhood cancer treatment.** Cumulative incidence is computed using the Aalen-Johansen estimator, while including death as a competing event. In our cohort of 7670 5-year CCS, the occurrence of a severe cardiac disease is highly influenced by the use of RT and CT for treating childhood cancer. 11% of patients treated with both RT and CT experience a severe cardiac disease within 50 years of their childhood cancer diagnosis, compared to only 2% of those treated without RT and CT. This is regardless of the occurrence of SMN. CCS: Childhood Cancer Survivors; CT: Chemotherapy; RT: Radiotherapy; SMN: Second Malignant Neoplasm.

# Additive Regression Model of the Cumulative Incidence of CD: Results


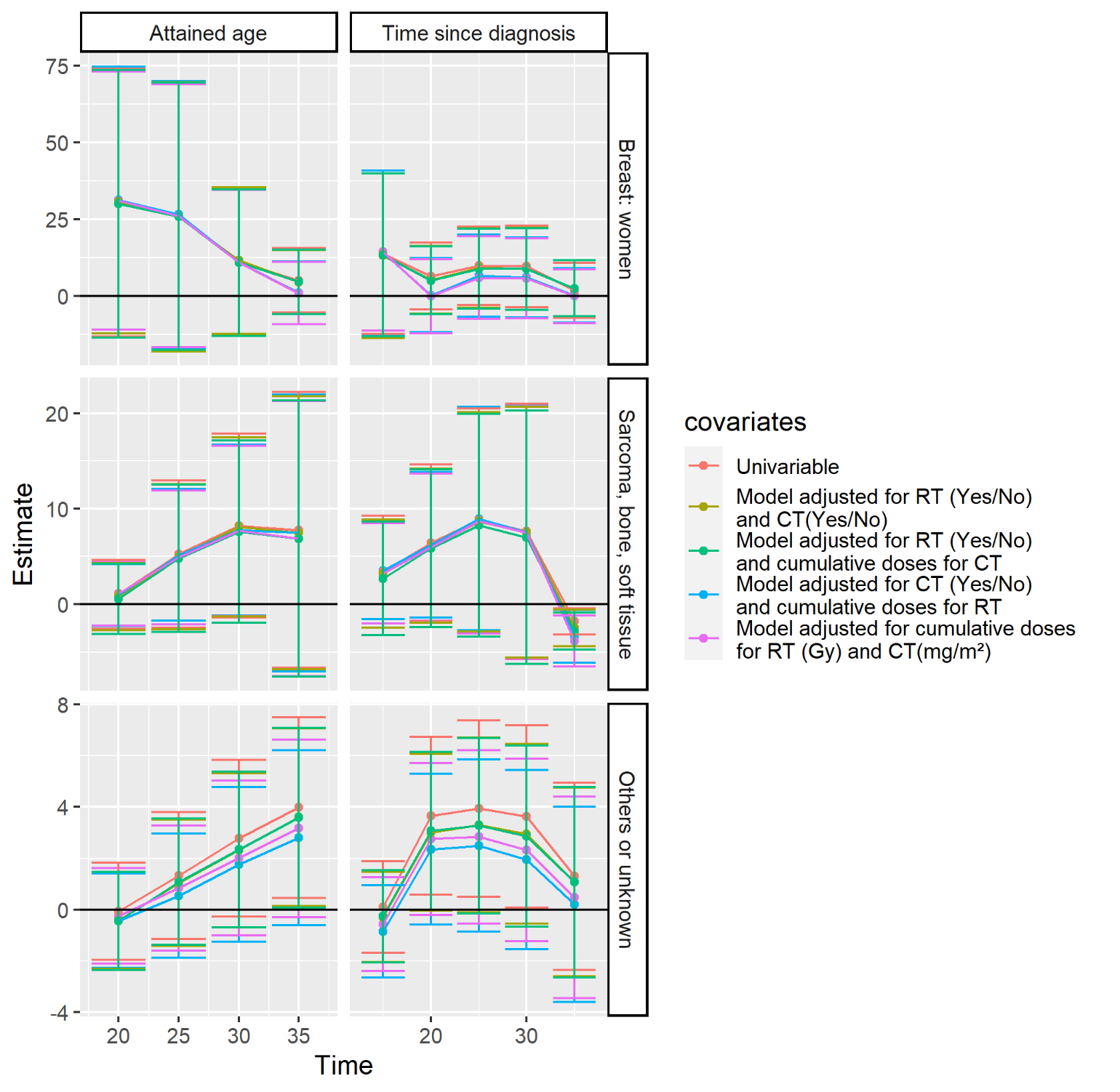


**Supplemental Figure 2**: **Additive effect of SMN on the Cumulative Incidence of Cardiac Disease.** We assigned patients experiencing a SMN to one of “Breast: women”, “Bone, Soft tissue, Sarcoma”, “Other or unknown” and compared them to those who had no SMN. We estimated breast cancer survivors diagnosed within 15 years of their childhood cancer diagnosis to have a cumulative incidence of cardiac disease 12.5% higher than childhood cancer survivors without SMN. The bands show the 95% confidence intervals. We can see that the confidence intervals are huge, and almost always include 0. However, also see that point-wise estimates are higher for sarcoma, bone, soft tissue, and breast cancer than for others. This is coherent with the cardiotoxicity associated with treatments of those cancers. Death was included as a competing event, and multivariable models were adjusted on sex, age at childhood cancer diagnosis, and year of childhood cancer diagnosis. SMN: Second Malignant Neoplasm.

# Unadjusted Cumulative Incidence


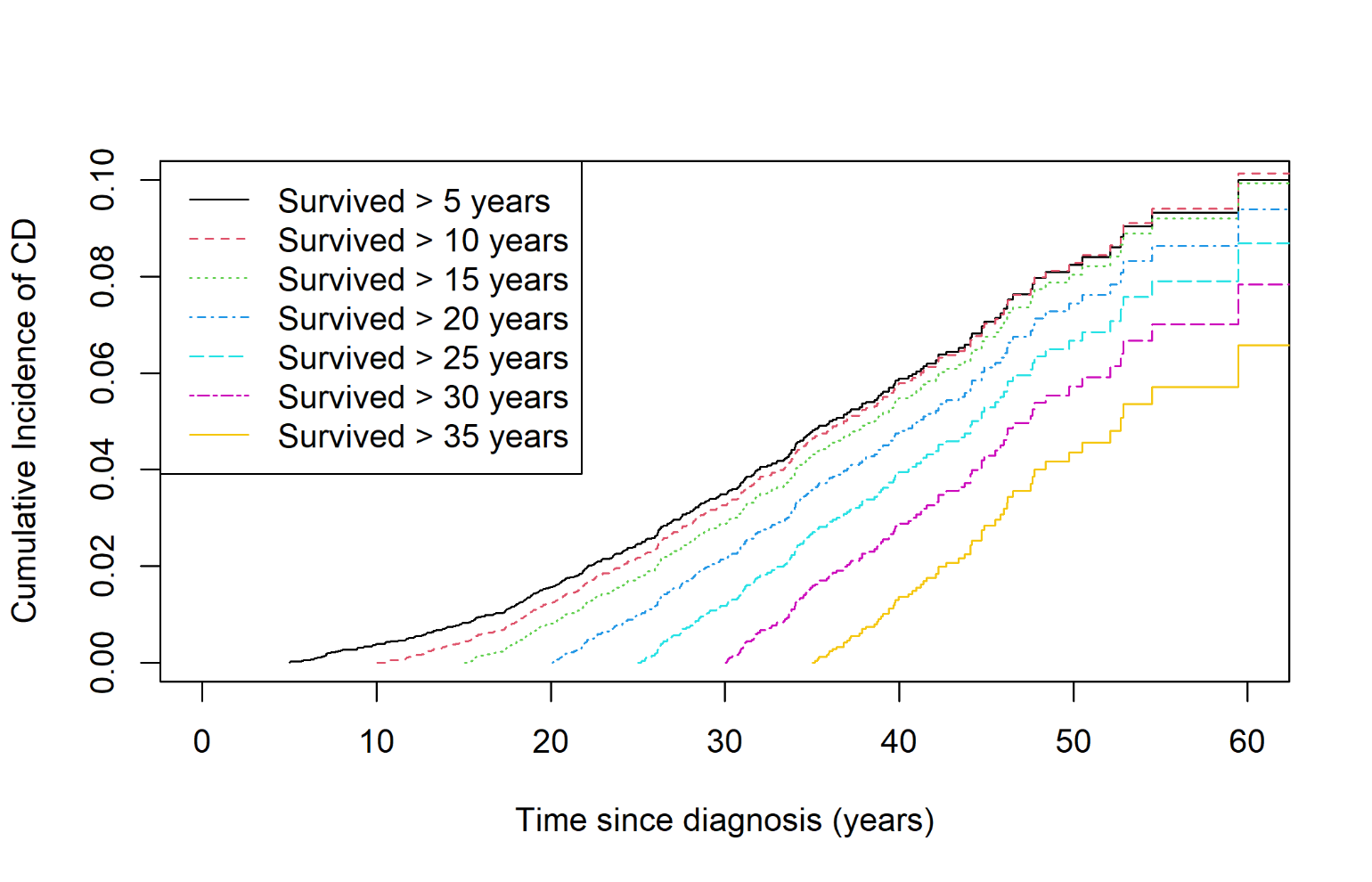


**Supplemental Figure 3**: **Cumulative Incidence of Cardiac Disease for CCS at each landmark time.** Cumulative incidence is computed using the Aalen-Johansen estimator, while including death as a competing event. We can see that no surge occurs, and every curve has a similar shape. Severe cardiac disease remains a rare event, with less than 10% of patients experiencing it within 60 years of childhood cancer diagnosis. CCS: Childhood Cancer Survivors

# Additive Regression Model of the Cumulative Incidence on the Competing Risk of Death: Results


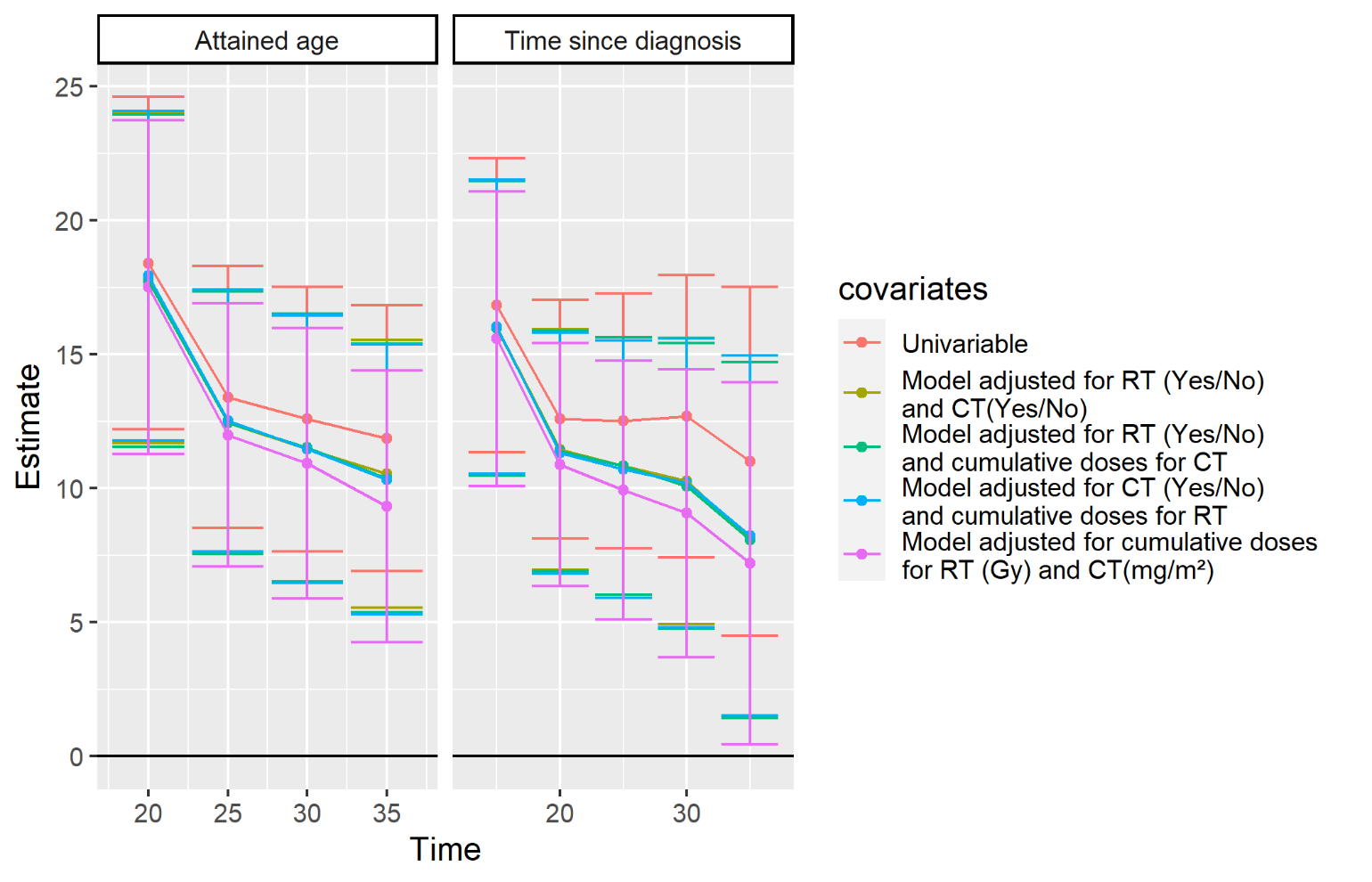


**Supplemental Figure 4**: **Additive effect of SMN on the Cumulative Incidence of death.** We used a landmark analysis, defining SMN as “experiencing any SMN before landmark time”, and estimated the effect of SMN on death. We used an additive model, and see that a SMN increases the cumulative incidence of death by more than 7.5% for all landmark times. Bands are 95% confidence intervals. We included cardiac disease and death by cardiac disease as competing events. Multivariable models were adjusted on sex, age at childhood cancer diagnosis, and year of childhood cancer diagnosis. SMN: Second Malignant Neoplasm.


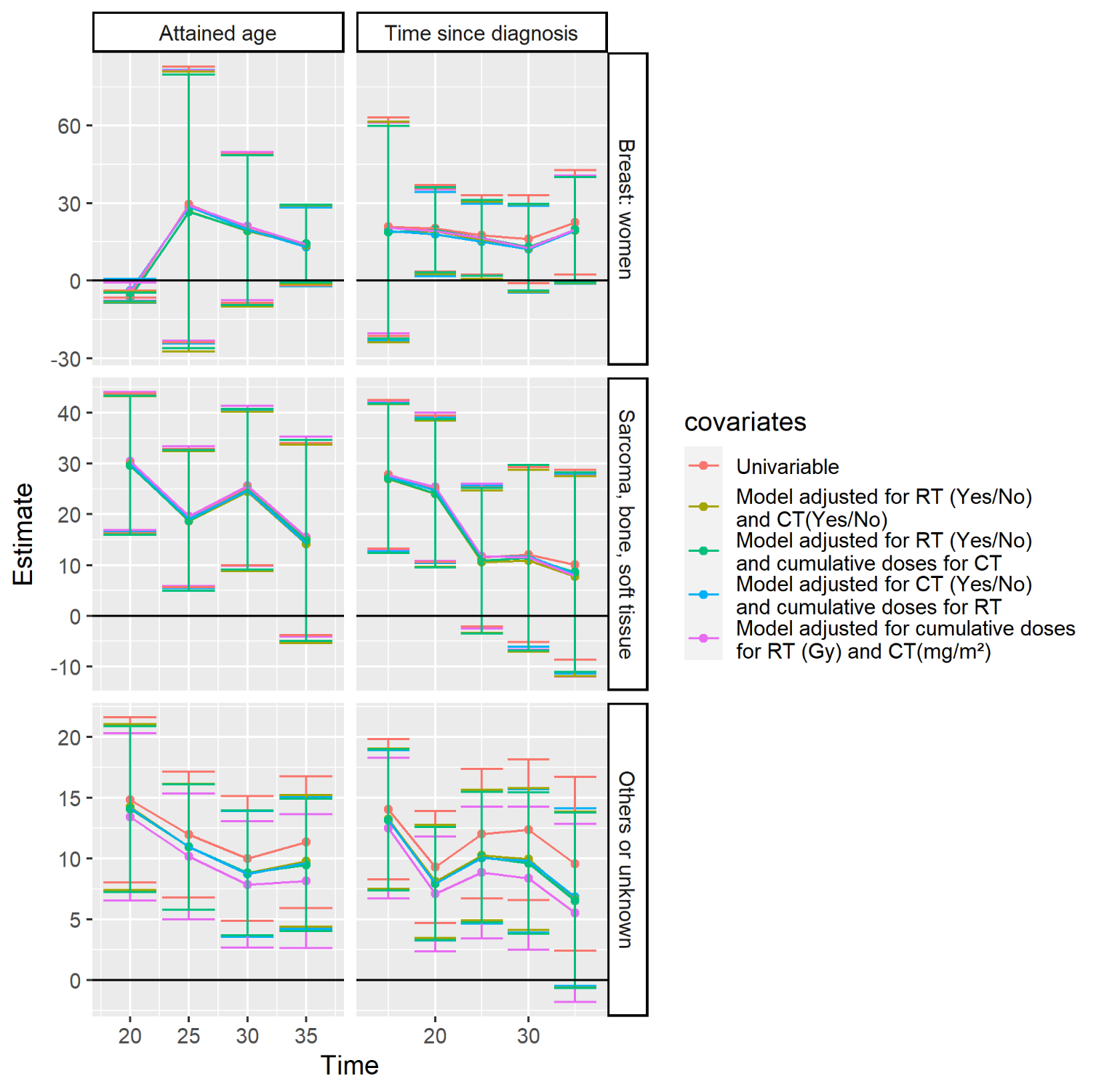
**Supplemental Figure 5**: **Additive effect of SMN on the Cumulative Incidence of death.** We used a landmark analysis, and assigned patients experiencing a SMN before landmark time to one of “Breast: women”, “Bone, Soft tissue, Sarcoma”, “Other or unknown” and compared them to those who had no SMN. We used an additive model to estimate the effect of SMN on death. We see that a SMN increases the cumulative incidence of death by more than 7.5% for all landmark times. Bands are 95% confidence intervals. We included cardiac disease and death by cardiac disease as competing events. Multivariable models were adjusted on sex, age at childhood cancer diagnosis, and year of childhood cancer diagnosis. SMN: Second Malignant Neoplasm

# Cumulative Incidence of Death


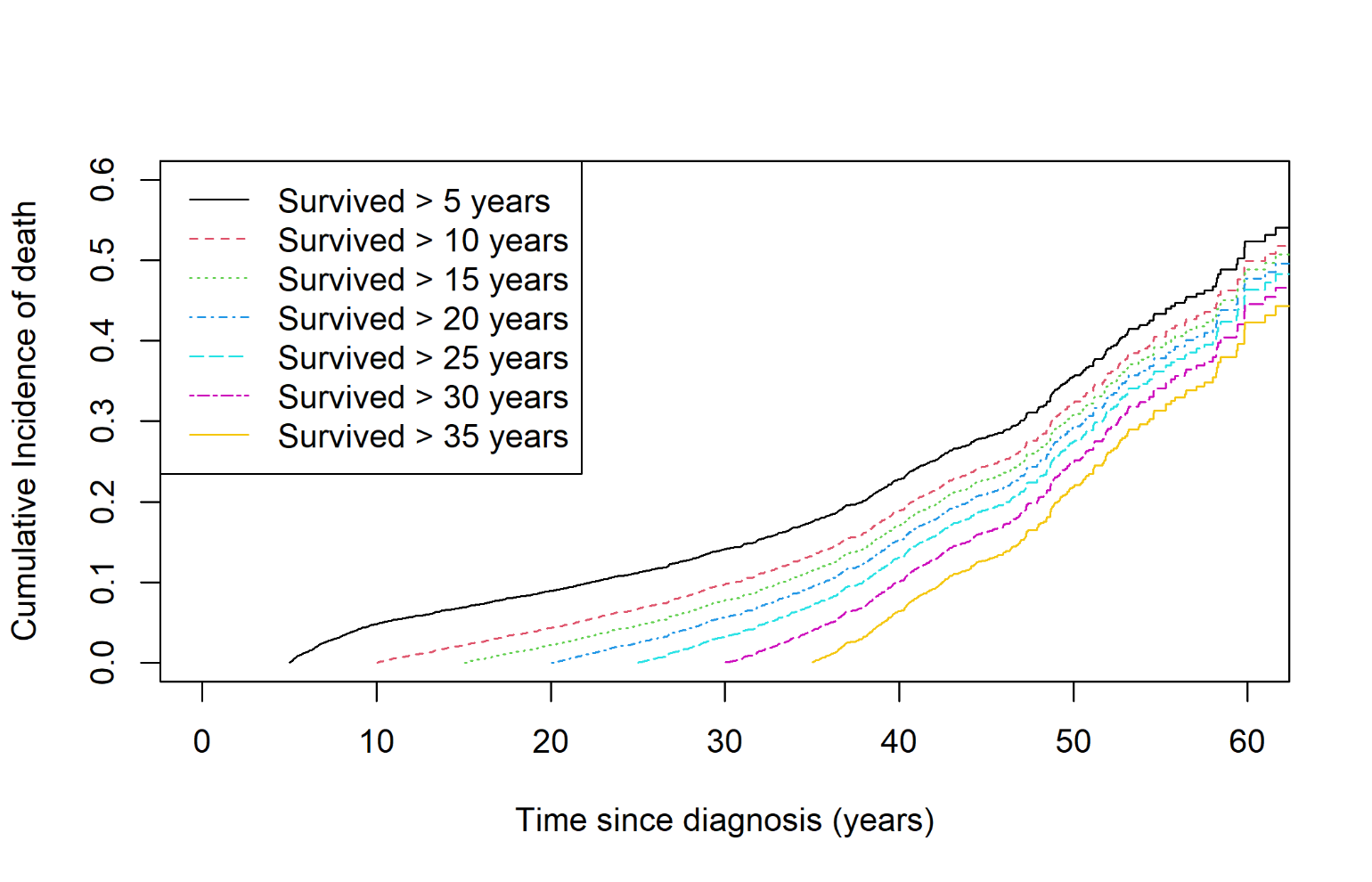


**Supplemental Figure 6**: **Cumulative Incidence of death for CCS at each landmark time.** Cumulative incidence is computed using the Kaplan-Meier estimator, without competing event. We can see that the rate of death is similar at each landmark time. This indicates that the patients included at each landmark time are roughly similar regarding their risk of death. CCS: Childhood Cancer Survivors.


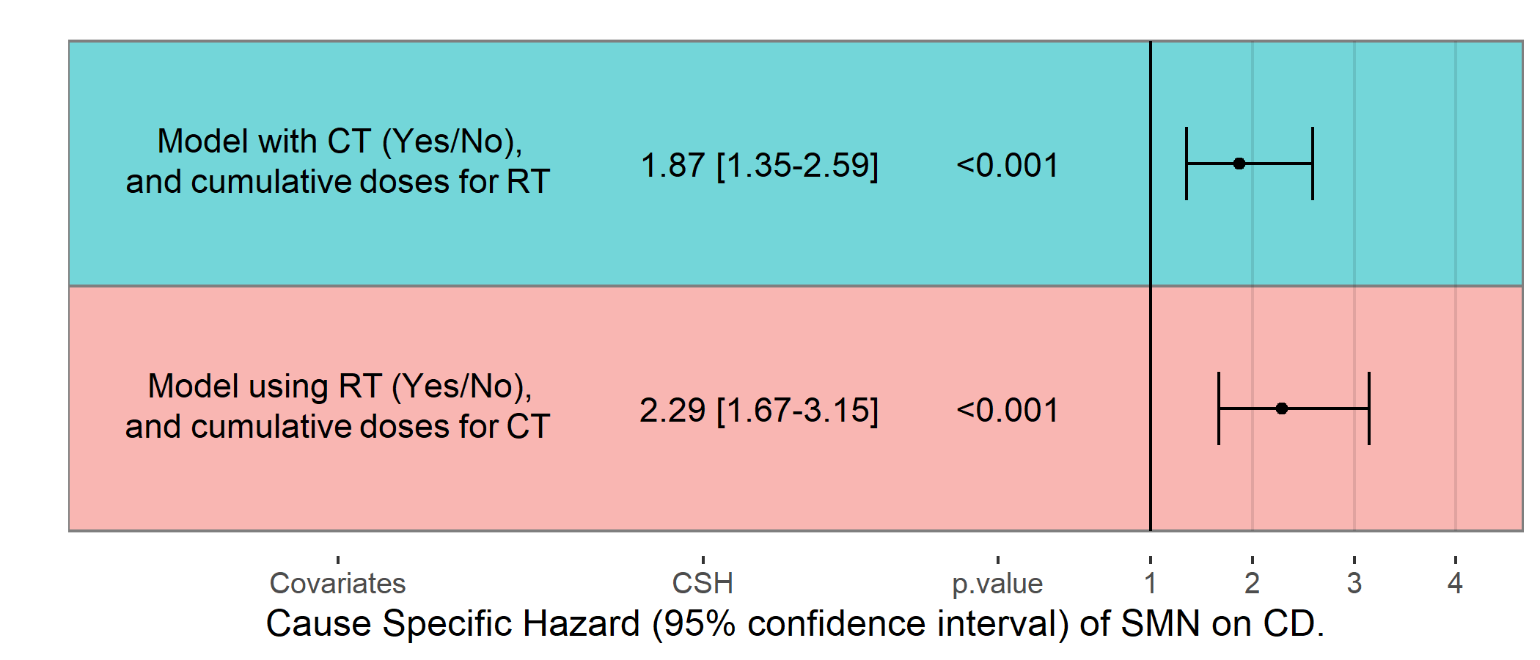


**Supplemental Figure 7: Multiplicative effect of SMN on the risk of cardiac disease.** Using a proportional cause specific hazard model, we found a 2-fold increase in the risk of cardiac disease after a SMN when adjusting on age at childhood cancer, sex, and either radiotherapy doses, chemotherapy doses, or both. The time-scale used for this analysis is “time since childhood cancer diagnosis”, and death is included as a competing event. Both models were adjusted on sex, age at childhood cancer diagnosis, and year of childhood cancer diagnosis. SMN: Second Malignant Neoplasm.

**
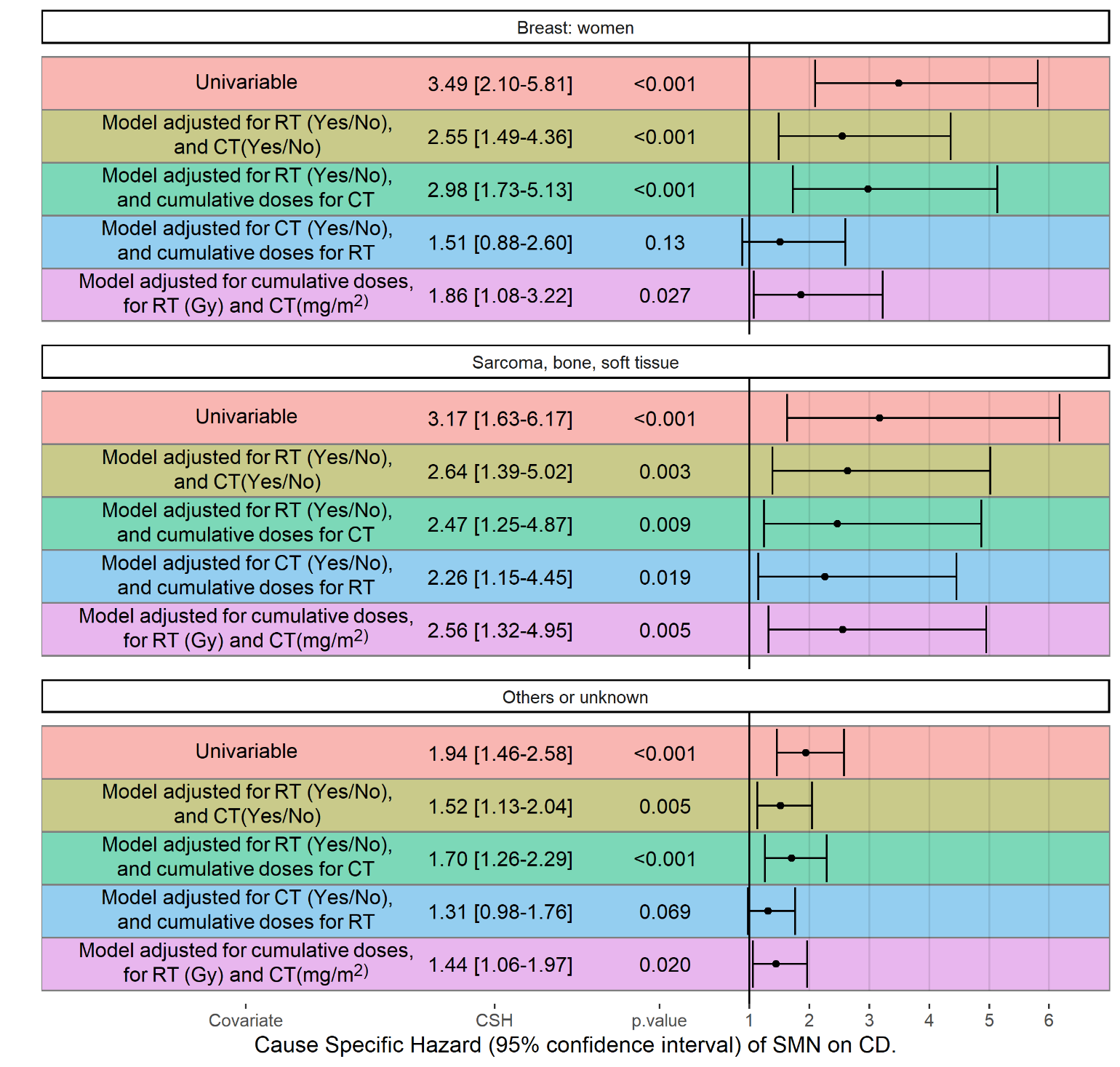
**

**Supplemental Figure 8**: **Multiplicative effect of SMN on the risk of cardiac disease .** We assigned patients experiencing a SMN to one of “Breast: women”, “Bone, Soft tissue, Sarcoma”, “Other or unknown” and compared them to those who had no SMN. Sarcoma, bone, and soft tissue cancer cause the highest increase, multiplying the risk by 2.37 compared to patients without SMN when adjusting on age at childhood cancer, sex, radiotherapy, and chemotherapy doses of childhood cancer treatment. The time-scale used for this analysis is “time since childhood cancer diagnosis”, and death is included as a competing event. Multivariable models were adjusted on sex, age at childhood cancer diagnosis, and year of childhood cancer diagnosis. SMN: Second Malignant Neoplasm.

# Cause Specific Hazard Model on the Competing Risk of Death: Results


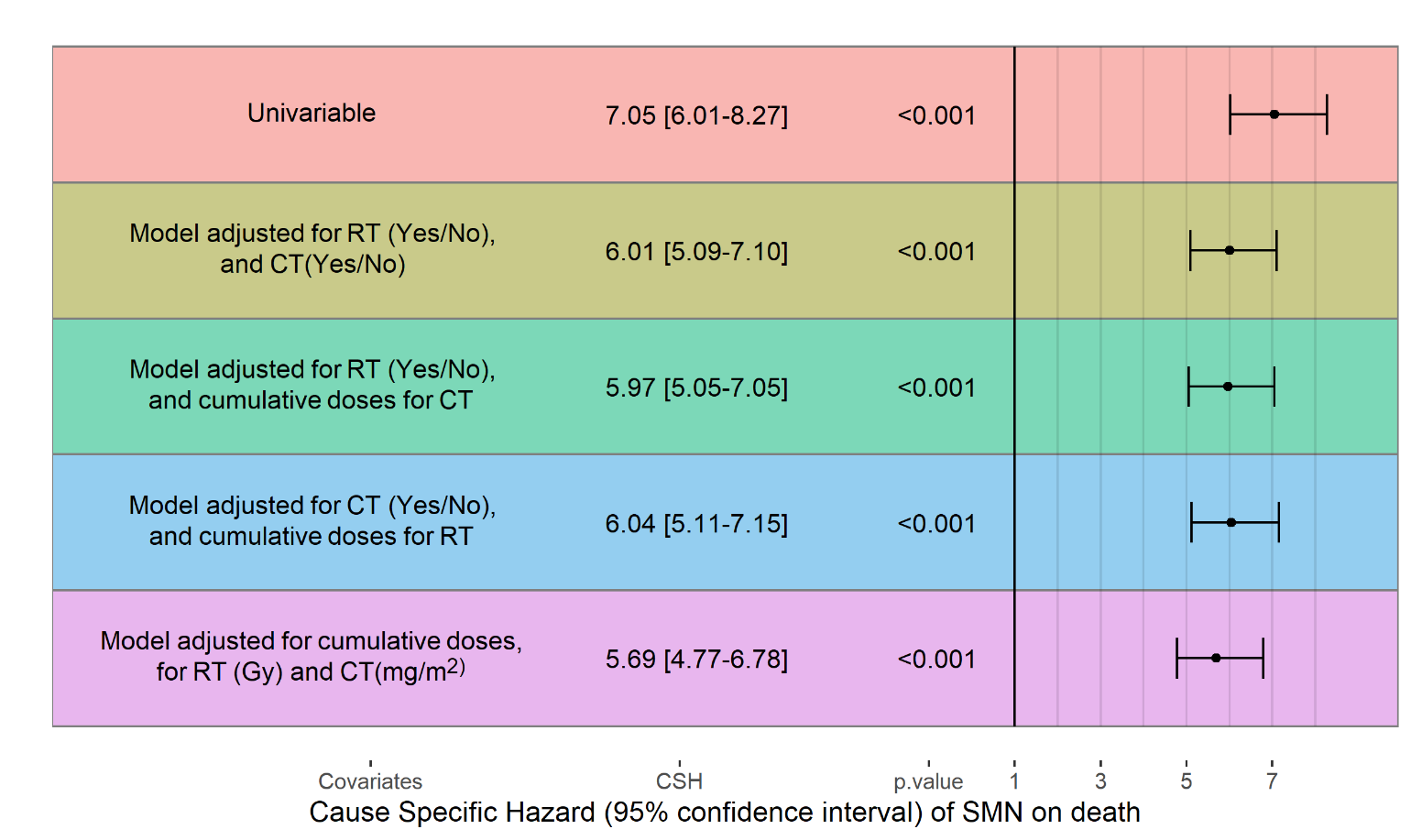


**Supplemental Figure 9**: **Multiplicative effect of SMN on the risk of death.** Using a proportional cause specific hazard model, we found a 5-fold increase in the risk of cardiac disease after a SMN when adjusting on age at childhood cancer, sex, and radiotherapy and chemotherapy doses used for childhood cancer treatment. The time-scale used for this analysis is “time since childhood cancer diagnosis”, and cardiac disease and cardiac disease -death are included as competing events. Multivariable models were adjusted on sex, age at childhood cancer diagnosis, and year of childhood cancer diagnosis. Those results show that including death as a competing event in the main analysis is required, and puts in perspective the 2-fold increase of cardiac disease risk. SMN: Second Malignant Neoplasm.


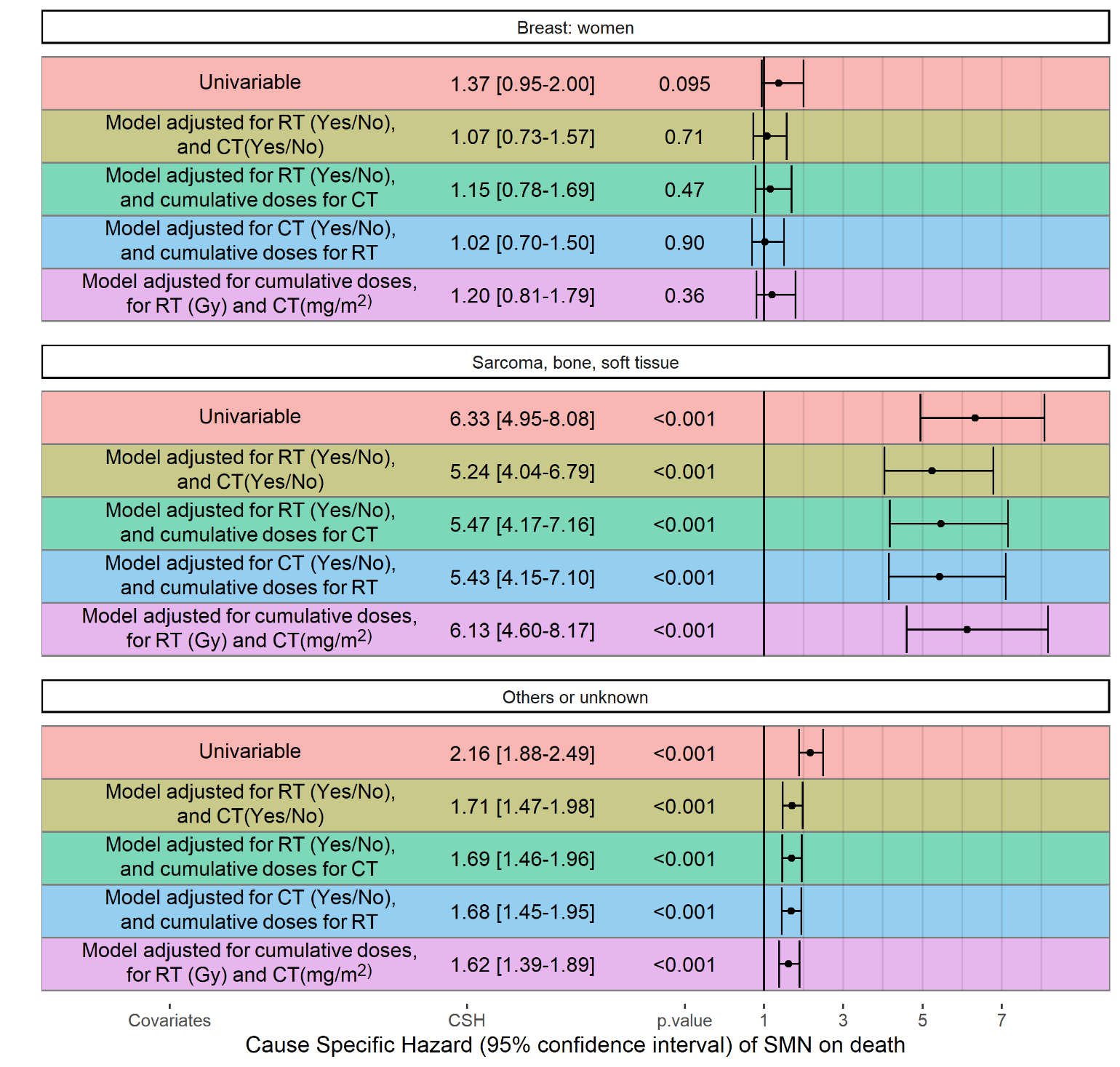


**Supplemental Figure 10**: **Multiplicative effect of SMN on the risk of death.** SMN is defined as one of “Breast cancer”, “Bone, Soft tissue, Sarcoma”, “Other or unknown” and the reference is “no SMN”. Using a proportional Cause Specific Hazard model, we found a 5-fold increase in the risk of cardiac disease after a SMN when adjusting on age at childhood cancer, sex, and radiotherapy and chemotherapy doses used for childhood cancer treatment. The time-scale used for this analysis is “time since childhood cancer diagnosis”, and cardiac disease and cardiac disease -death are included as competing events. Multivariable models were adjusted on sex, age at childhood cancer diagnosis, and year of childhood cancer diagnosis. Those results show that including death as a competing event in the main analysis is required, and puts in perspective the 2-fold increase of cardiac disease risk. SMN: Second Malignant Neoplasm.

# Times of SMN by Landmark Time


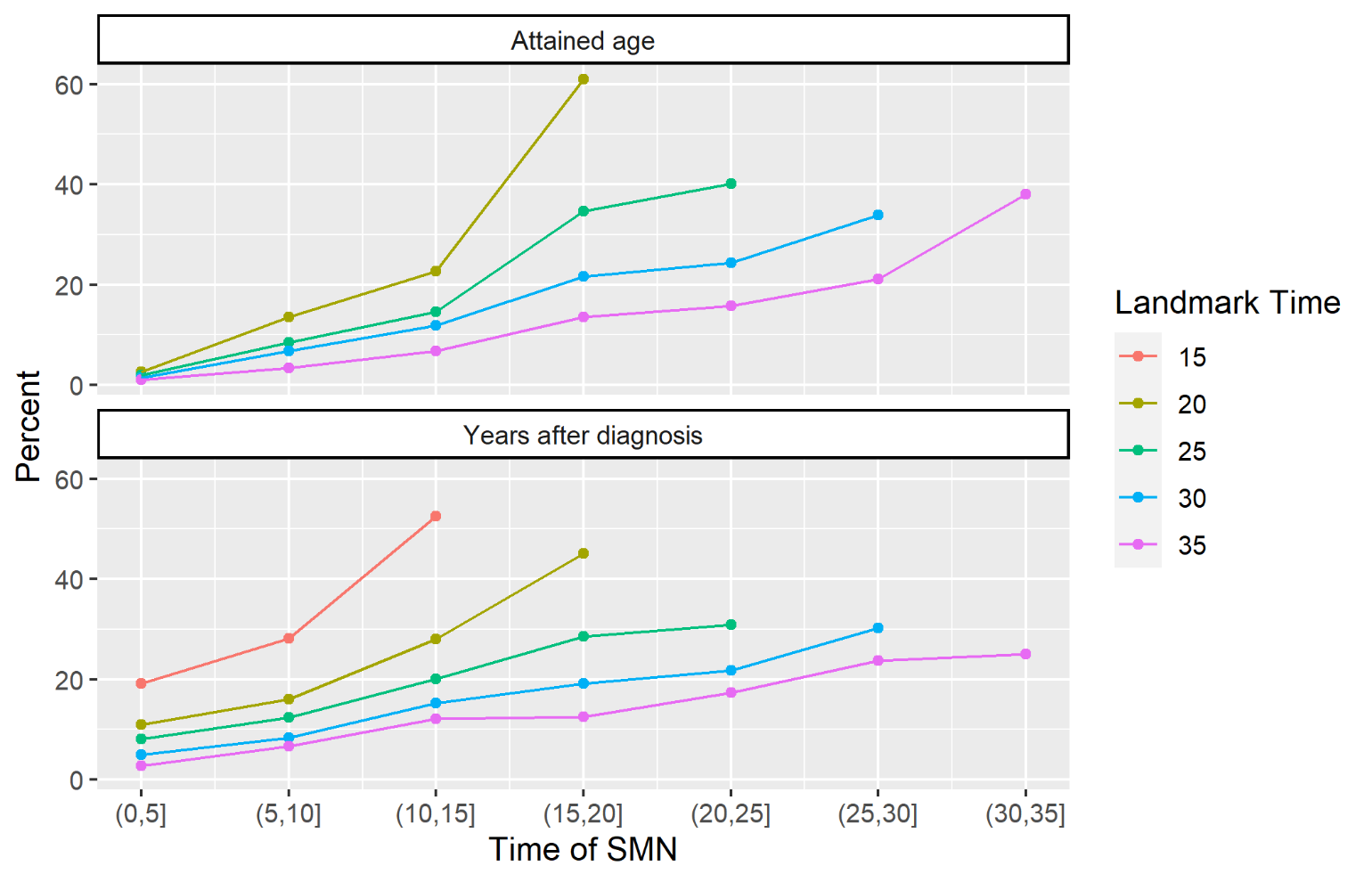


**Supplemental Figure 11**: **Distribution of SMN times for each landmark time.** For each time point used for the landmark analysis, shows the repartition across time of SMN occurrences. Only patients who are still at risk at landmark time are included. Landmark analysis does not take into account the effect of time since SMN diagnosis, so this raw description brings helpful context to the main results. We can see that patients recently diagnosed (<5 years) account for 25 to 60 percent of the population depending on landmark time. This is important to understand, because it helps understand the huge effect of SMN on death at first landmark times. Landmark time is expressed in years. SMN: Second Malignant Neoplasm.
